# Hypothesis‐Driven Research on Multiple Stressors: An Analytical Framework for Stressor Interactions

**DOI:** 10.1002/ece3.71959

**Published:** 2025-08-12

**Authors:** Iris Madge Pimentel, Dania Albini, Arne J. Beermann, Florian Leese, Samuel J. Macaulay, Christoph D. Matthaei, James A. Orr, Jeremy J. Piggott, Ralf B. Schäfer

**Affiliations:** ^1^ Aquatic Ecosystem Research University of Duisburg‐Essen Essen Germany; ^2^ School of Life Sciences University of Essex Colchester UK; ^3^ Centre for Water and Environmental Research (ZWU) University of Duisburg‐Essen Essen Germany; ^4^ Department of Biology University of Oxford Oxford UK; ^5^ Department of Zoology University of Otago Dunedin New Zealand; ^6^ School of the Environment University of Queensland Brisbane Queensland Australia; ^7^ Discipline of Zoology and Trinity Centre for the Environment Trinity College Dublin Dublin 2 Ireland; ^8^ Research Centre One Health Ruhr and Faculty of Biology, Ecotoxicology University of Duisburg‐Essen Essen Germany

**Keywords:** co‐tolerance, cumulative effects, generalized regression models, multiple stressors, null‐model testing, statistical interaction, stressor interaction

## Abstract

Identifying and characterizing stressor interactions is central to multiple stressor research. Such interactions refer to stronger (synergism) or weaker (antagonism) joint effects of co‐occurring stressors on biological entities, when compared to the predictions of a theoretical null model. Various null models have been developed, and the selection of the most appropriate null model for a specific research question is ideally based on assumptions on co‐tolerance patterns in communities and mechanisms of stressor effects. Statistical models are commonly used to evaluate the statistical significance of interaction terms. However, they introduce constraints by imposing a specific null hypothesis on stressor combinations that cannot be flexibly changed. This can introduce a mismatch between the null model that the analyst wants to test and the one imposed by the statistical model. Here, we show under which conditions the statistical null hypothesis for interaction terms misaligns with a multiple‐stressor null model and propose to resolve such misalignments using post‐estimation inference. Null‐model specific interaction estimates can be calculated from adjusted predictions of a fitted regression model, and associated standard errors are derived using the delta method, posterior simulations, or bootstrapping. We illustrate the suggested approach with three case studies and validate statistical conclusions through data simulations. Post‐estimation inference has the potential to advance hypothesis‐driven research on stressor interactions by flexibly testing any a priori defined null model independent from regression model structure.

## Graphical abstract

Multiple‐stressor research is relevant across ecological sub‐disciplines (terrestrial, marine, and freshwater), aiming to predict how anthropogenic stressors combine. Different null models have been developed for this purpose. Here, we introduce an analytical framework to statistically evaluate any a priori chosen null model to facilitate hypothesis‐driven research on multiple stressors.

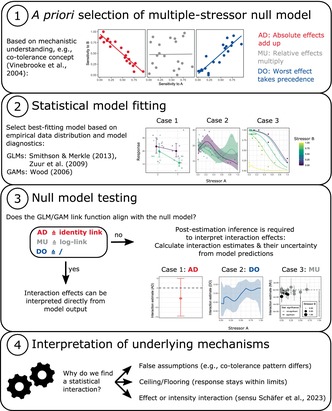

## 1 Introduction

Ecosystems worldwide are exposed to rapid environmental change and to an increasing number and concentration of pollutants due to human activities. We refer to these anthropogenic alterations as “stressors” if an environmental parameter is pushed outside of its natural variability and affects individuals, populations, biotic communities, or ecosystem functioning (sensu Piggott, Townsend, and Matthaei 2015). The co‐occurrence of two or more stressors is common (Halpern et al. 2008; Ormerod et al. 2010; Rillig et al. 2023; Schäfer et al. 2016; Waite et al. 2021), and human resource exploitation will likely exacerbate this problem. Consequently, multiple‐stressor research has been growing in importance across ecological subdisciplines, such as terrestrial, marine, or freshwater ecology (Orr et al. 2020). Such research aims to advance our ability to predict the cumulative net effect of co‐occurring stressors based on their individual effects by using a null model that mathematically describes how stressor effects combine (Folt et al. 1999; Schäfer and Piggott 2018). However, empirical observations of net effects often deviate from predictions based on individual stressor effects, and stressors are therefore inferred to interact (e.g., Crain et al. 2008; Darling and Côté 2008; Holmstrup et al. 2009; Jackson et al. 2016; Morris et al. 2022; Silva et al. 2002; Thrupp et al. 2018). The characterization and mechanistic explanation of these stressor interactions is one of the primary goals of multiple‐stressor research.

Formulating clear a priori hypotheses about the mechanisms by which stressors and their combinations affect a biological response is necessary to improve predictions of ecological change (Griffen et al. 2016; Schäfer and Piggott 2018). The selection of an appropriate null model is crucial in multiple‐stressor studies because the chosen null model establishes our *null hypothesis* on how stressors combine. It is contrasted with the *alternative hypothesis* of a stressor interaction, ideally accompanied by a mechanistic explanation (e.g., warming may intensify the exposure of aquatic organisms to toxicants, because it increases metabolic activity). Two null models commonly applied in ecology are the simple addition and multiplicative null models, which assume that the absolute effects of the individual stressors add up or the relative effects of the stressors multiply, respectively (Folt et al. 1999). Folt et al. (1999) also discuss the dominance null model, which predicts that the net effect becomes the single “worst effect of the individual stressors”. More complex models such as the concentration addition (Loewe and Muischnek 1926) or the stress addition null model (Liess et al. 2016) have been developed in (eco)toxicology to predict the effect of toxicant mixtures or of single toxicants combined with environmental stressors (see Schäfer et al. 2023). The predictions of these different null models can strongly diverge (Dey and Koops 2021; Morris et al. 2022). Consequently, while one null model may make predictions equivalent to empirical observations, another one may overestimate, and a third one may underestimate the biological response to stressor combinations. Therefore, the detection of an interaction, its classification as synergism (stronger net effect than predicted) or antagonism (weaker net effect than predicted) and the magnitude of the interactive effect depends on null model choice, and a meaningful discussion of an interaction requires explicit reference to the tested null model's mechanistic assumptions.

When identifying a stressor interaction, statistical tools can determine if collected empirical data give sufficient evidence to reject the null hypothesis. This raises the question: Which analytical approach can be used for which null model? In factorial experiments, we expose our focal organisms or communities to two or more stressors, such that control conditions without any stressor, single‐stressor exposures, and all possible stressor combinations are tested (e.g., Arias Font et al. 2021; Beermann et al. 2018; Bruder et al. 2016; Davis et al. 2019; Elbrecht et al. 2016; Juvigny‐Khenafou et al. 2021b). Data are then commonly analyzed with multifactorial analysis of variance (ANOVA), while experiments or field studies exploring gradients of stressor intensity (e.g., Lemm and Feld 2017; Lourenço et al. 2023; Madge Pimentel et al. 2024; Piggott, Niyogi, et al. 2015; Wagenhoff et al. 2012) are often analyzed with multiple linear regression models. These approaches allow testing the effect of two or more predictors on the biological response and specify statistical interaction terms, which represent the focal stressors and their interactions, respectively. Using untransformed response data imposes testing the simple addition null model, while the same approach for logarithmically transformed responses imposes the multiplicative null model (Duncan and Kefford 2021; Schäfer and Piggott 2018; Spake et al. 2023).

Importantly, every statistical model makes assumptions about data properties. The central assumptions of the general linear model include that the response variable is normally distributed for each predictor combination, with a mean conditional on the predictors and a shared variance that is constant across treatments (Ståhle and Wold 1989). Biological responses, however, often correspond to count data (e.g., species richness or population abundances) and follow a Poisson distribution, or they correspond to proportions (e.g., survival rate or remaining leaf mass as a measure of organic matter decomposition) and follow a binomial or beta distribution, each of which implies a non‐constant mean–variance relationship and therefore violates the model assumptions. Therefore, data transformations are commonly performed to improve the fit of the biological response to the required assumptions (see Feld et al. 2016). Such transformations may result in a misalignment between the intended and actual null model being tested. In fact, Griffen et al. (2016) found that approximately a third of marine multiple‐stressor studies reviewed in Crain et al. (2008) used logarithmic transformations, yet discussed stressor interactions as if the underlying null model was additive. However, this issue is not trivial to mitigate. The unintentional evaluation of a different null model as a consequence of data transformations can lead to erroneous conclusions (Spake et al. 2023); yet, a violation of the assumptions of the statistical model can also distort these conclusions (Zuur et al. 2010). A considerable part of the reported context‐dependency of stressor effects may be an artifact of methodological differences in the statistical analyses of multiple‐stressor studies (Catford et al. 2022). Therefore, a unifying framework for the formal analysis of stressor interactions that resolves the problems addressed above is urgently needed.

**FIGURE 1:**
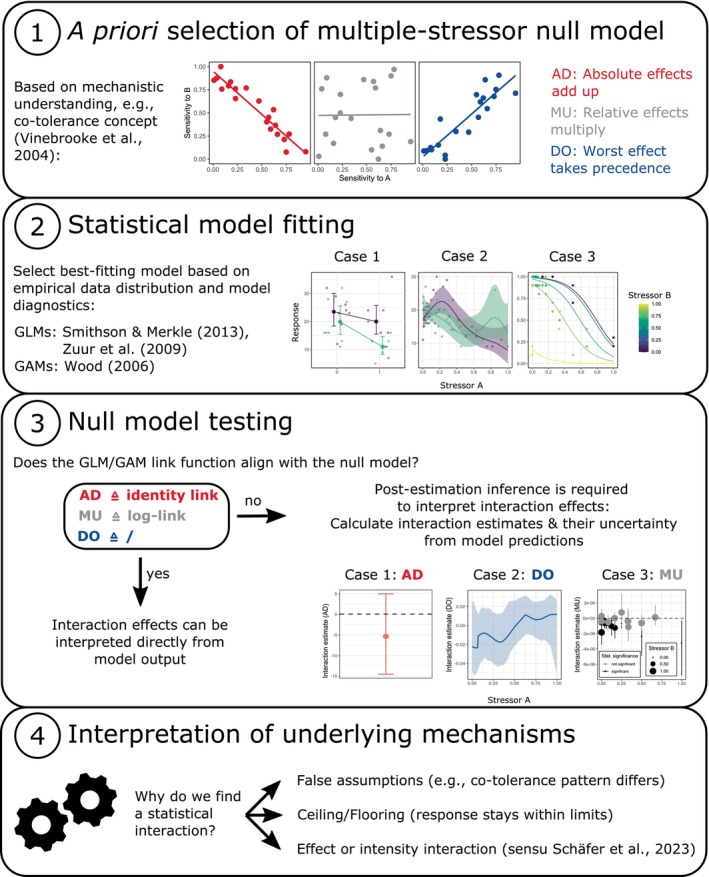
Analytical framework for stressor interactions. With our proposed approach, researchers (1) select a null model a priori based on ecological theories, (2) specify the best‐fitting statistical model for the data, (3) test the selected null model with post‐estimation inference, and (4) discuss the underlying mechanisms for detected deviations from the null model.

Here, we briefly review the current state of knowledge about different multiple‐stressor null models and present an analytical framework that enables ecologists to explicitly evaluate an a priori chosen null model. After discussing key considerations for null model selection, we demonstrate the limitations of using interaction terms in generalized linear models (GLMs) and their equivalents in generalized additive models (GAMs) to test for stressor interactions. Instead, we propose calculating null‐model‐specific interaction estimates and their statistical uncertainty from a fitted GLM/GAM. In brief, to allow for high flexibility in a null model choice, our analytical framework separates the process of statistical model fitting from the process of null model hypothesis testing (Figure 1). We use three empirical datasets with different experimental designs as case studies to showcase the application of our approach for multiple‐stressor studies and validate its robustness with data simulations. These datasets include an experimental design with two categorical stressors (case study 1), a design with one categorical stressor and one continuous stressor (case study 2) and a design with two continuous stressors (case study 3, Figure 1).

## 2 Multiple‐Stressor Null Models and Their Mechanistic Assumptions in the Co‐Tolerance Framework

In applied ecology, the simple addition null model (AD) forms the basis for the most commonly used stressor interaction classification systems (e.g., Crain et al. 2008; Piggott, Townsend, and Matthaei 2015). It assumes that the stressors' individual effects add up. If *C*, *S*_*A*_, and *S*_*B*_ denote the biological response for control and stressor conditions A and B, respectively, we can define our prediction for the combined stressor response (*S*_*A,B*_) as:

(1) $$S_{A,B}=S_{A}+S_{B}-C$$

Even if not explicitly stated, a null interaction (i.e., conformity with null‐model predictions), synergism, and antagonism are conventionally interpreted with this null model in mind. However, this convention does not mean that the AD frequently generates accurate predictions. In fact, it is prone to making implausible predictions for most biological responses, because they can typically not fall below zero (e.g., population size, biomass, and species richness). This natural limit on the net effect is easily crossed when adding up single‐stressor effects. In these cases, a deviation from the AD does not imply any mechanistic interaction between stressors.

Nevertheless, before reaching the effect limit, it can make sense to assume simple addition. The co‐tolerance concept (Vinebrooke et al. 2004) illustrates this and offers a useful framework for null‐model selection when two stressors affect species richness. Vinebrooke et al. (2004) showed that a strong negative correlation between stressor sensitivities suggests the choice of the AD as the null model because we expect completely different subsets of species to be affected by each stressor. If stressor sensitivities are uncorrelated, the multiplicative null model (MU) is most appropriate (Vinebrooke et al. 2004) and assumes that the relative effects of the stressors multiply. As a result, it accounts for “double hits”, i.e., for species which would be driven to local extinction by each of the stressors:

(2) $$S_{A,B}=\frac{S_{A}\times S_{B}}{C}$$

In the case of a positive sensitivity correlation, both stressors affect the same subset of species, and the “worst” single‐stressor effect is expected to take precedence (Vinebrooke et al. 2004). This corresponds to the dominance null model (DO):

(3) $$\min\left(C,S_{A,B}\right)=\min\left(S_{A},S_{B}\right)$$

Note that our mathematical definition differs from previous formulations of the DO, in which the response under joint stressor exposure is either predicted to take the lowest value observed (Schäfer and Piggott 2018) or the value that deviates strongest from control conditions (Morris et al. 2022). The motivation for our adjusted definition is to allow for the two stressors to have positive or opposing effects on the response. Changes in environmental variables such as temperature, salinity, or oxygen concentrations are likely to be detrimental to some species, but either directly or indirectly beneficial for others. This has been taken into account to refine stressor‐interaction classification systems (Piggott, Townsend, and Matthaei 2015) but has often been neglected in the context of the co‐tolerance framework. The term “stressor” is misleading in these cases because an increase in stressor intensity releases the focal species from physiological stress or adverse biotic interactions, causing better performance and survival. Here, we conceptualize “stressor” as a driver of biological change irrespective of effect direction and formulate the DO in a way that it consistently corresponds to positively correlated sensitivities (Data S1).

The co‐tolerance framework can also be applied to population sizes of a single species, where certain phenotypes may show negatively or positively correlated sensitivities to two stressors (Jarvis et al. 2024). Therefore, we can explain deviations from the MU by sensitivity correlations; if these deviations are located within the margins defined by the AD and DO. At first sight, this seems not to imply true independence of stressors; however, the *physiological* stressor effects within each organism may still act independently. Therefore, AD can be used to test for stressor interactions that cannot be explained by negatively correlated sensitivities, and the DO can be used to test for stressor interactions that cannot be explained by positively correlated stressor sensitivities. This approach can be suggestive of alternative explanations such as metabolic trade‐offs (e.g., Liess et al. 2016; Moe et al. 2013) or cross‐talk and cross‐tolerance (Sinclair et al. 2013; Velasco et al. 2019) between physiological pathways mediating stressor tolerance.

### 2.1 Limitations of the Co‐Tolerance Framework

**FIGURE 2:**
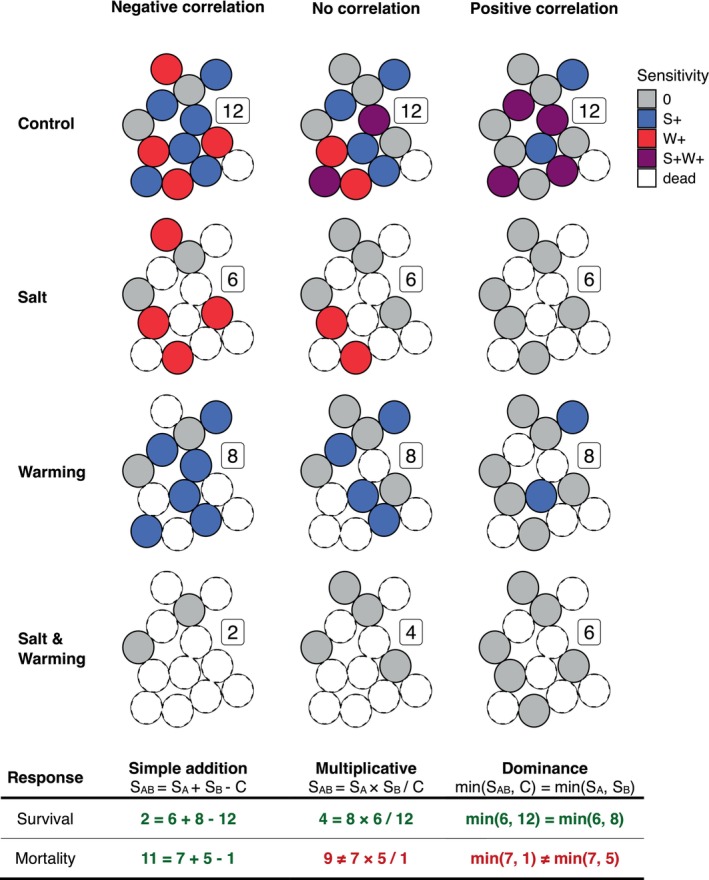
Applicability of multiple‐stressor null models in the co‐tolerance framework using survival or mortality for prediction of stressor net effects. Three co‐tolerance scenarios (negatively, positively correlated or uncorrelated sensitivities) are explored. Filled circles represent survivors; empty circles represent individuals that have died. Colors indicate the sensitivity to salinization (S+) and warming (W+, see figure legend). Except for simple addition, the null models make asymmetric predictions that differ between the response metrics (survival vs. mortality) and only make predictions concordant with the co‐tolerance framework if survival is chosen over mortality.

When applied to continuous metrics such as biomass, it is not evident how stressors combine at the individual level. The co‐tolerance framework provides an assessment of the combined effects based on the correlation between the tolerances to the individual stressors. Application of this framework to count‐based metrics (survival/death, stay/emigrate…) is straightforward. However, stressor‐induced changes in continuous metrics such as biomass might follow different rules when being combined at the individual level rather than at the population level. Even if we assume that the same null model used at the population level (e.g., MU if we assume uncorrelated sensitivities) applies to individual‐level biomass changes, predictions based on population‐level responses can still differ from those obtained by aggregating individual‐level stressor responses. This is because measurements collected at the community or population level can mask the variation at the individual level (see Data S1.2). To make accurate quantitative predictions, therefore, the co‐tolerance framework requires individuals or species to respond in a binary pattern (e.g., persistence or extinction, stay or emigrate) resulting in discrete or proportional metrics such as abundances, richness, or number of survivors. Nevertheless, for binary responses it is not always evident what we should consider to be the response. For example, we can either quantify the number of individuals which have died after stressor application or the number of individuals that survived. The AD makes symmetric predictions, irrespective of our choice for the response metric, i.e., analyses can be based on the number of survivors or the number of dead specimens. The MU and DO, however, make asymmetric predictions, i.e., the predictions calculated from the number of survivors differ from those calculated from the number of dead specimens and only the former correspond to the co‐tolerance concept (Figure 2). It is important to recognize that our null model definitions only reliably represent uncorrelated or positively correlated sensitivities if an increase in the response corresponds to tolerance (e.g., survival rate, number of individuals staying) and its decline to sensitivity toward the treatment conditions. Moreover, the requirement of binary individual responses restricts the applicability of the co‐tolerance framework to discrete biological responses such as counts, while continuous data (e.g., total population biomass, growth, and respiration rates) may frequently be of interest but do not satisfy this condition.

### 2.2 Multiple‐Stressor Null Models Beyond the Co‐Tolerance Framework

The AD, MU, and DO are all commonly used in ecological studies on combinations of environmental stressors. However, ecotoxicological research on toxicant mixtures has developed a different, yet overlapping set of null models to predict the net effect of two or more toxicants (Schäfer et al. 2023). Although not discussed in detail here, these ecotoxicological null models can also be useful for environmental stressors and complement the co‐tolerance concept (see Schäfer and Piggott 2018). For example, the stress addition model (Liess et al. 2016) was developed to predict the increase in an organism's sensitivity to a toxicant when an environmental stressor exerts additional metabolic stress (Liess et al. 2016). Moreover, the effects of toxicants that have analogous modes of action, i.e., the biological mechanism through which the toxicant effect manifests, are expected to combine according to the concentration addition model (CA, Loewe and Muischnek 1926). In ecotoxicology, detailed mode‐of‐action classification systems have been developed to facilitate the assessment of the applicability of CA and describe where (molecular target site) and how (physiological response) a toxicant affects the organism (Kienzler et al. 2017; Martin et al. 2013). Although environmental stressors may also act sufficiently similar to justify the use of the CA, mode‐of‐action categorizations for stressors in ecology are still underdeveloped and only distinguish broadly between stressors acting destructively on the whole organism (e.g., flash‐flood), on specific physiological pathways (e.g., pesticides), and generally on physiology (e.g., warming; Schäfer and Piggott 2018). A more nuanced view based on dynamic energy budget theory may further facilitate null‐model choice (Simmons et al. 2021) by asking if the physiological stressor effect is on growth, maintenance, reproduction, feeding, or a combination of those responses (see Galic et al. 2018).

## 3 Statistical Evaluation of Stressor Interactions

Analytical frameworks for stressor interactions have been developed based on fixed cutoff values for departure from null‐model predictions (Belden et al. 2007; Tekin et al. 2020). However, this approach does not consider the uncertainty of empirical observations. Pronounced differences between null‐model predictions and actual observations can easily arise by chance if sample sizes are small (Macacu and Guillot 2020). Below, we discuss commonly used statistical tools in multiple‐stressor studies and explain under which conditions they fail in testing stressor interactions. Focusing on the three null models embedded within the co‐tolerance framework (AD, MU and DO), we propose an alternative statistical approach, post‐estimation inference, which is established for interaction analyses in medical and socio‐political research disciplines (Ai and Norton 2003; McCabe et al. 2022; Mize et al. 2019; VanderWeele and Knol 2014).

### 3.1 Interaction Terms in Regression Models and Their Relation to Multiple‐Stressor Null Models

Ecological studies often use regression models, in particular generalized linear models (GLMs) and generalized additive models (GAMs), as their analytical foundation (Feld et al. 2016; Kefford et al. 2023; Pedersen et al. 2019). These statistical models enable the simultaneous assessment of the effects of multiple predictors on the biological response. The predictors are also called explanatory or independent variables and include our primary predictors (e.g., the two focal stressors) and covariates, which may be needed to account for confounding effects (e.g., by unintentional differences in water temperature among pond mesocosms). Depending on our experimental design, we can specify numerical predictors that cover a stressor gradient, or factorial stressors that may be binary (control condition vs. stressor condition), ordered (multiple levels of increasing stressor values, but differences between stressor levels are not consistent in scale), or nominal (different categories without any intrinsic order). The popularity of GLMs, particularly for multiple‐stressor studies, is likely rooted in the ease with which interaction terms between two or more predictors can be incorporated into the model's equation. These interaction terms are then often directly interpreted in the context of stressor interactions.

Frequently, analysis of variance (ANOVA) has been used because the majority of multiple‐stressor experiments apply factorial stressor designs (Orr et al. 2024). ANOVA can be viewed as a subclass of a GLM that assumes a Gaussian distribution of the response and has only categorical predictors (O'Hara 2009). However, ecological data are unlikely to meet core assumptions of ANOVA: Within each treatment group, the response must be normally distributed, and the mean–variance relationship is expected to be constant. For count data, for example, this is not the case. They should follow a Poisson distribution, which implies that the variance within a treatment group increases with higher average counts. To avoid violations of ANOVA's assumptions, the analyst may stabilize the variance by transforming the data, using, for example, a logarithmic transformation. However, data transformations change the interpretation of interaction terms in regression models: While an ANOVA with untransformed data evaluates the AD, a logarithmic transformation imposes the MU as the multiple‐stressor null model (Duncan and Kefford 2021; Griffen et al. 2016; Spake et al. 2023). Moreover, data transformations introduce bias to the model's predictions on the response scale (Warton et al. 2016; Zuur et al. 2010), which can be avoided by specifying a GLM with a suitable conditional distribution (O'Hara and Kotze 2010).

**FIGURE 3:**
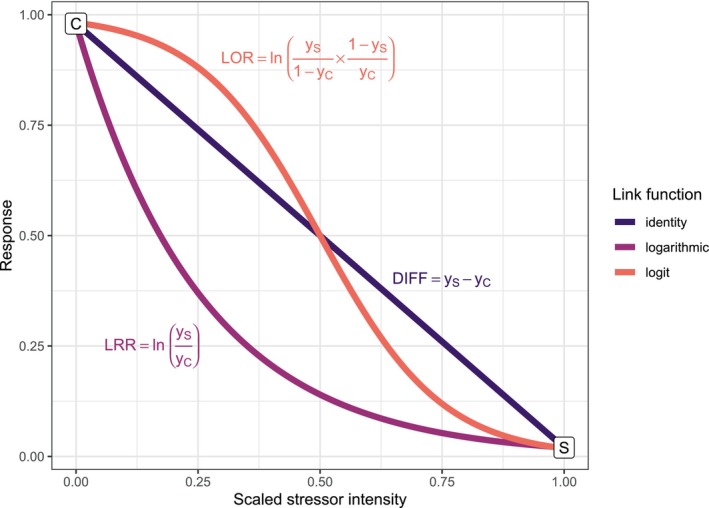
Link functions and their implications for stressor–response relationships and effect measures. Under control conditions (stressor intensity = 0, point C), a response of 98% is observed; for maximum stressor conditions (stressor intensity = 1, point S), a response of 2% is observed. For the identity link, we obtain a linear stressor–response relationship, and the effect measure is the difference between stressor and control conditions (DIFF). For the logarithmic link, we obtain an exponentially decreasing stressor–response relationship, and the effect measure is the log response ratio (LRR). For the logit link, we obtain a sigmoidal stressor–response relationship, and the effect measure is the log odds ratio (LOR).

GLMs can be used to evaluate non‐normally distributed data without data transformations. Nevertheless, the interpretation of interaction terms in GLMs can suffer from similar complications as their interpretation after data transformation. To comprehend this, we must familiarize ourselves with the regression equation and link functions. GLMs have three components that the analyst can specify (McCullagh and Nelder 1998): (1) The conditional distribution of the response variable, (2) the systematic part, and (3) the link function. While the conditional distribution determines the mean–variance relationship for the response variable, the systematic part and the link function determine the regression equation. In the systematic part, we specify our primary predictors, covariates and interaction terms. The link function connects this systematic part with the expected value or mean of the response variable (μ). In doing so, it determines the form of the predictor–response relationship that the GLM can model, if predictors are gradients (Figure 3). Depending on the conditional distribution of our GLM, there will be a “canonical” link function that simplifies the algorithmic procedure of model fitting and ascertains that the model's predictions stay within the natural limit of the response (e.g., above 0 for counts in Poisson regression, or between 0 and 1 for proportions in binomial and beta regression; McCullagh and Nelder 1998). For Gaussian regression models, the canonical link is the identity link, which results in linear stressor–response relationships. For Poisson regression, on the other hand, the logarithmic link is typically used and imposes an exponential increase or decrease. Binomial and beta regression for proportional data use a logit‐link function that imposes a sigmoidal, i.e., S‐shaped curve (Figure 3).

(4) $$\text{link}\left(\mu \right)=\beta_{0}+\beta_{1}S_{A}+\beta_{2}S_{B}+\beta_{3}S_{A}S_{B}$$

In this example, we want to investigate how stressor A ($S_{A}$) and B ($S_{B}$) jointly affect the biological response. The regression model estimates four coefficients, including the intercept (β_0_), the stressors' main effects (β_1_ and β_2_), and the interactive effect (β_3_). The intercept describes the expected response for control conditions on the scale of the link function, when both stressors are set to zero. The main effects describe how much the response changes on the link scale as we move from control to stressor conditions for factors, or as we increase the stressor intensity by one unit for stressor gradients (e.g., by 1°C for temperature). The interaction effect describes how much the main effect of A on the link scale changes for each unit increase in stressor intensity of B, and vice versa. Ideally, this quantity represents the stressor interaction we are interested in. However, as outlined below, this is not necessarily the case because the interpretation of the coefficients depends on the link function.

This can be shown by formulating the null hypothesis associated with the interaction term. The statistical tests used for the main and interaction terms in regression models (e.g., Wald test, F‐tests, or likelihood ratio test) fundamentally evaluate the null hypothesis that a particular model coefficient is equal to 0 (Zuur et al. 2009). For the interaction coefficient, this means we test:

(5) $$H_{0}\left(\beta_{3}=0\right):\text{link}\left(\mu \right)=\beta_{0}+\beta_{1}S_{A}+\beta_{2}S_{B}$$

To comprehend the implications of a link function for this null hypothesis, we must apply its inverse to both sides of the equation. This translates the hypothesis from the link scale to the response scale. We base our example on the logarithmic link and exponentiate both sides of the equation:

(6.1) $$\ln\left(\mu \right)=\beta_{0}+\beta_{1}S_{A}+\beta_{2}S_{B}$$

(6.2) $$\mu =e^{\beta_{0}+\beta_{1}S_{A}+\beta_{2}S_{B}}=e^{\beta_{0}}\times e^{\beta_{1}S_{A}}\times e^{\beta_{2}S_{B}}$$

It becomes evident that the addition of stressor effects on the logarithmic scale becomes something different on the response scale: the statistical model now assumes that single‐stressor effects multiply. Moreover, by imposing a different stressor–response relationship, the link function also changes the interpretation of the main effects. With the identity link, β_1_ and β_2_ describe the absolute change in the response caused by each of the stressors. With the logarithmic link, it describes the multiplicative change, i.e., the ratio between control and stressor conditions. Consequently, the term “effect” is ambiguous and can refer to a variety of effect measures such as differences, ratios, or odds ratios (Figure 3). In summary, the link function determines (1) the effect measure that the model coefficients describe, (2) the form of the predictor–response function for gradients, and (3) the statistical model's null hypothesis on how predictors combine mathematically in the absence of an interactive effect.

For gradients, the predictor–response function may, however, take a shape unknown to the analyst. In this case, we can use GAMs, which extend the toolbox of GLMs by fitting smooth curves that flexibly accommodate a variety of nonlinear predictor–response functions (Wood 2006). The popular *mgcv* package (version 1.9‐1, Wood 2011) for the R programming environment (version 4.4.1, R Core Team 2024) can be used for fitting GAMs. It implements automatic smoothness selection to avoid overfitting and facilitates the familiar ANOVA decomposition into main effects and interaction effects with tensor product and factor‐smooth interactions. In these GAMs, the link function no longer enforces a specific stressor–response relationship, while still determining the null hypothesis for stressor combinations. Therefore, if we have enough sampling points along the gradient(s), GAMs can be a useful tool for multiple‐stressor analysis. For more detailed information on GAM theory, we refer the interested reader to the documentation of the *mgcv* package (Wood 2023) and to Wood (2011), Wood (2006), as well as Pedersen et al. (2019).

Theoretically, we can now address a misalignment between the canonical link function of our GLM or GAM and the multiple‐stressor null model we want to test by the specification of the appropriate noncanonical link function (i.e., identity link for the AD, and logarithmic link for the MU). For example, Bentley et al. (2022) review approaches for interaction analysis on response variables that are probabilities and suggest the application of a binomial model with identity link to test for interactions on the response scale, and a log‐binomial model for multiplicative interactions. However, regression models specified this way frequently fail to converge (VanderWeele and Knol 2014). This is because probabilities drawn from a binomial distribution are confined to the interval between 0 and 1, and while the canonical link of binomial regression, i.e., the logit link, ensures that model predictions cannot fall outside this range, the identity and logarithmic link do not. Moreover, the confidence intervals can become biased because they no longer reflect the skewness of the underlying distribution. In addition, the approach is restricted to testing the AD or MU, as the DO does not align with any link function. Instead, we suggest separating the process of statistical model specification from that of hypothesis testing (see also Mize et al. 2019; Warton et al. 2016). Model diagnostics, which are described in detail elsewhere (e.g., Smithson and Merkle 2013; Wood 2006; Zuur et al. 2009), and a priori knowledge on data properties are used to find the most appropriate model structure. The fitted and validated regression model can then be used to test hypotheses on stressor combinations based on the model's adjusted predictions.

### 3.2 Post‐Estimation Inference to Resolve Misalignments Between Interaction Terms and Multiple‐Stressor Null Models

We can calculate estimates for stressor interactions for the AD (${\hat{\mathrm{int}}}_{\mathrm{AD}}$), MU (${\hat{\mathrm{int}}}_{\mathrm{MU}}$), and DO (${\hat{\mathrm{int}}}_{\mathrm{DO}}$) based on the null model definitions above. A detailed derivation of the definitions below from Equations (1) to (3) is given in Data S1. We first defined $f\left(a,b|\overset{⃑}{x}\right)$ as the conditional function that describes the biological response on the response scale and is taken from the fitted regression model. It is dependent on our two focal stressors a and b, while all other covariates are held constant at values specified in the vector $\overset{⃑}{x}$. With this function, we predict the response for all combinations of our two focal stressors given the factor levels (or values for a gradient) considered as control ($a=c_{A}$ and $b=c_{B}$) or stressor conditions ($a=s_{A}$ and $b=s_{B}$). This is how we can calculate our interaction estimate for the AD:

(7) $${\hat{\mathrm{int}}}_{\mathrm{AD}}∶=\frac{f\left(s_{A},s_{B}|\overset{⃑}{x}\right)-f\left(c_{A},s_{B}|\overset{⃑}{x}\right)-f\left(s_{A},c_{B}|\overset{⃑}{x}\right)+f\left(c_{A},c_{B}|\overset{⃑}{x}\right)}{\left(s_{A}-c_{A}\right)\times \left(s_{B}-c_{B}\right)}$$

The term in the denominator is a rearranged version of Equation (1), while the nominator standardizes the interaction estimate by the amount of change in our predictors between stressor and control conditions. This becomes important for stressor gradients. For factorial stressors, the term $s_{A}-c_{A}$ (and/or $s_{B}-c_{B}$) is set to 1. With this definition of the interaction estimate, our null hypothesis when using the AD can be mathematically described as ${\hat{\mathrm{int}}}_{\mathrm{AD}}=0$. Similarly, we can formulate definitions for interaction estimates for the MU and DO. Predictions from the fitted regression model are logarithmically transformed for ${\hat{\mathrm{int}}}_{\mathrm{MU}}$, so that the null hypothesis can be defined as ${\hat{\mathrm{int}}}_{\mathrm{MU}}=0$:

(8) $${\hat{\mathrm{int}}}_{\mathrm{MU}}∶=\frac{\ln\left[f\left(s_{A},s_{B}|\overset{⃑}{x}\right)\right]-\ln\left[f\left(c_{A},s_{B}|\overset{⃑}{x}\right)\right]-\ln\left[f\left(s_{A},c_{B}|\overset{⃑}{x}\right)\right]+\ln\left[f\left(c_{A},c_{B}|\overset{⃑}{x}\right)\right]}{\left(s_{A}-c_{A}\right)\times \left(s_{B}-c_{B}\right)}$$

(9) $${\hat{\mathrm{int}}}_{\mathrm{DO}}∶=\frac{\min\left[f\left(s_{A},s_{B}|\overset{⃑}{x}\right),f\left(c_{A},c_{B}|\overset{⃑}{x}\right)\right]-\min\left[f\left(c_{A},s_{B}|\overset{⃑}{x}\right),f\left(s_{A},c_{B}|\overset{⃑}{x}\right)\right]}{\left(s_{A}-c_{A}\right)\times \left(s_{B}-c_{B}\right)}$$

For binary factorial stressors, the selection of control and stressor conditions is arbitrary and only changes the direction of the interaction estimates (positive or negative). For stressor gradients, we suggest using the observed stressor values as control conditions and a small increase in stressor intensity (e.g., 0.01% of the stressor's range) as stressor conditions when calculating the interaction estimates. In doing so, we approximate the slope of the stressor–response function at the observed stressor value, either on the response scale (AD and DO) or on the logarithmic scale (MU). For the AD and MU, we essentially calculate (1) the change in the effect of one stressor when the second stressor comes into play for factor–factor combinations, (2) the change in slope between control and stressor conditions of the other stressor for factor–gradient combinations, or (3) the cross derivative of the joint stressor–response surface for gradient–gradient combinations (see also Ai and Norton 2003; McCabe et al. 2022; Mize et al. 2019; Onukwugha et al. 2015). For the DO, we calculate the absolute difference between stressor and control conditions (or the slope) for the weaker stressor, given that the stronger stressor already exerts an effect.

**FIGURE 4:**
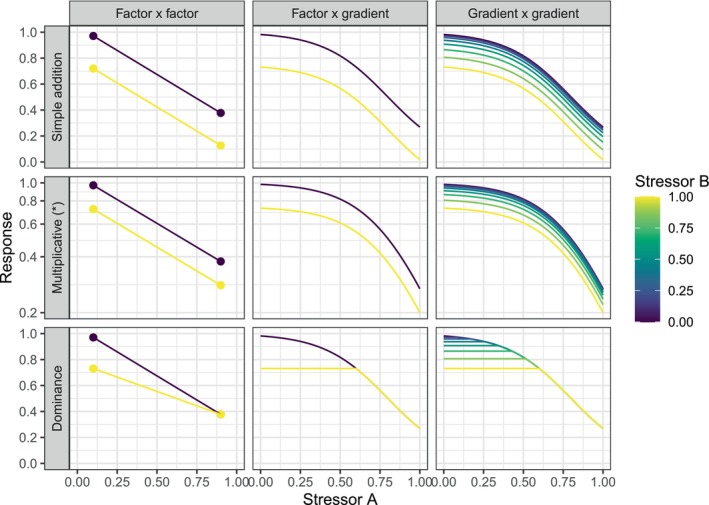
Graphical representation of multiple‐stressor null models for factor–factor, factor–gradient, and gradient–gradient combinations. (*) Note that the y‐axis is logarithmically transformed for the multiplicative null model.

A graphical representation of the three null models for factor–factor, factor–gradient, and gradient–gradient combination may clarify these concepts. For this purpose, we can use conditional plots, i.e., we plot the stressor–response relationship of stressor A given the different values of stressor B (Figure 4). When this is done on the scale of interest, the visual interpretation of these plots also helps us in categorizing and describing the stressor interaction. For the AD, we expect parallel stressor–response functions for the different strata of the other stressor on the response scale (Figure 4). For the MU, we expect the same on the logarithmic scale (Figure 4). In both cases, synergistic interactions would be expressed as a steeper slope for higher values of the other stressor, and an antagonistic interaction would be expressed as a flatter slope. More nuanced categorizations exist for factorial stressors (Piggott, Townsend, and Matthaei 2015). For the DO, visual interpretation is independent of the scale used for plotting. Here, we expect the stressor–response relationship of the stronger stressor to be identical (not only parallel) for different values of the weaker stressor. Because the stressor ranking may change across a gradient, we can expect to observe flat sections if the focal stressor is weaker in that section, and a sudden change in slope as the stressor becomes the stronger one (Figure 4). As discussed by Spake et al. (2023), it is advisable to explore conditional plots symmetrically, i.e., by plotting each of the two focal stressors on the x‐axis and using the other for faceting.

It is important to note that the interaction estimates are dependent upon all predictors included in the regression model. They may not only change along the stressor gradients but also between different sets of covariates. Above, we noted that these covariates are held constant at values specified by the analyst. We may choose specific values, such as the means or modes of the covariates (“interaction at the mean”) or other values of interest (“interaction at representative values”), such as optimistic and pessimistic levels of a third stressor to inform ecological management decisions (Stoffels and White 2024). Alternatively, we can use the observed values. By calculating the interaction estimates for every observation, we can use their mean as a single summary statistic (“average interaction”), which gives a good representation for the interaction in our sample (McCabe et al. 2022). In our case studies presented below, we calculate these average interaction estimates and explore graphically how observation‐wise interaction estimates change across the predictors.

The calculation of the interaction estimates requires combining the coefficients of the regression model nonlinearly. Therefore, the associated standard errors must be approximated after model estimation to construct confidence intervals. Asymptotic standard errors can be calculated in different ways (Dowd et al. 2014), including the delta method (Ai and Norton 2003), simulations based on the posterior distribution of the model coefficients (K‐R method, Krinsky and Robb 1986), or nonparametric methods such as bootstrapping (Efron and Tibshirani 1998). The delta method is the most commonly used approach, as it is computationally efficient and produces similar estimates for the standard error compared to simulation‐based approaches or bootstrapping (Dowd et al. 2014). However, it assumes that the function used to combine the model coefficients is “locally linear” and continuously differentiable (Dowd et al. 2014). This is not the case for the DO, where abrupt changes occur (Figure 4). In practice, standard errors computed by the delta method will be inaccurate close to these abrupt changes. Therefore, in the case studies below, we use the K‐R method for uncertainty estimation.

In summary, the analytical strategy we suggest involves (1) fitting (and validating) a GLM or GAM with the appropriate conditional distribution, (2) calculating the average interaction estimate of interest (${\hat{\mathrm{int}}}_{\mathrm{AD}}$, ${\hat{\mathrm{int}}}_{\mathrm{MU}}$ or ${\hat{\mathrm{int}}}_{\mathrm{DO}}$) across observations, while holding covariates at their observed values, (3) constructing confidence intervals around this average interaction estimate using the K‐R method, and (4) interpreting the interaction graphically by plotting observation‐wise interaction estimates with their standard errors, and adjusted model predictions. Below, we exemplify this analysis with three case studies, including a factor–factor, a factor–gradient, and a gradient–gradient combination of two stressors. We employed the R programming environment for our analyses (version 4.4.1, R Core Team 2024), using the packages *mgcv* for GAMs (version 1.9–3, Wood 2023), *DHARMa* (version 0.4.6, Hartig 2022) for regression model diagnostics, and *marginaleffects* (version 0.27.0, Arel‐Bundock et al. 2024) to calculate interaction estimates and their uncertainty. The R code to reproduce our analyses is provided along with two custom‐made functions to facilitate the analysis of specific null models in Data S2 and in our OSF data repository (https://osf.io/J4DBZ/).

## 4 Case Studies

### 4.1 Case Study 1: Factor–Factor Interaction

Beermann et al. (2018) conducted a full‐factorial stream mesocosm experiment (*N* = 64) to investigate how macroinvertebrate communities respond to salinization (ambient salinity: 18.2 ± 4.1 [SD] mg/L chloride, increased salinity: 312.2 ± 78.5 mg/L chloride), fine sediment addition (low sediment cover: 9.4% ± 1.7%, high sediment cover: 81.8% ± 9.8%), reduction in flow velocity (control flow velocity: 16.5 ± 0.1 cm/s, reduced flow velocity: 9.6 ± 0.1 cm/s), and combinations thereof. Here, we focus on analyzing how salinity and flow velocity affect abundances of mayfly nymphs in the family Baetidae. Baetid species are sensitive to both of the focal stressors (Beermann et al. 2018; Cañedo‐Argüelles et al. 2012; Juvigny‐Khenafou et al. 2021a; Szöcs et al. 2012). However, the physiological mechanisms through which the stressors affect the organisms are distinct: Slow flow velocities reduce oxygen availability (Pardo and García 2016), while salinization damages tissue through localized salt poisoning and increases metabolic costs for osmotic regulation (Kefford 2019). Due to the difference in physiological mechanisms, we postulate negatively correlated sensitivities and use the AD for prediction.

Because baetid abundances were over‐dispersed (see Data S2), we specified a negative binomial regression model with a logarithmic link function of the form:

(10) $$\begin{array}{cc} \ln\left(\text{Abund}\right)= & \beta_{0}+\beta_{1}\mathrm{Sa}+\beta_{2}\mathrm{Se}+\beta_{3}\mathrm{Fl}+\beta_{4}\text{SaSe}+\beta_{5}\\ & \text{SaFl}+\beta_{6}\text{SeFl}+\beta_{7}\text{SaSeFl} \end{array}$$

**FIGURE 5:**
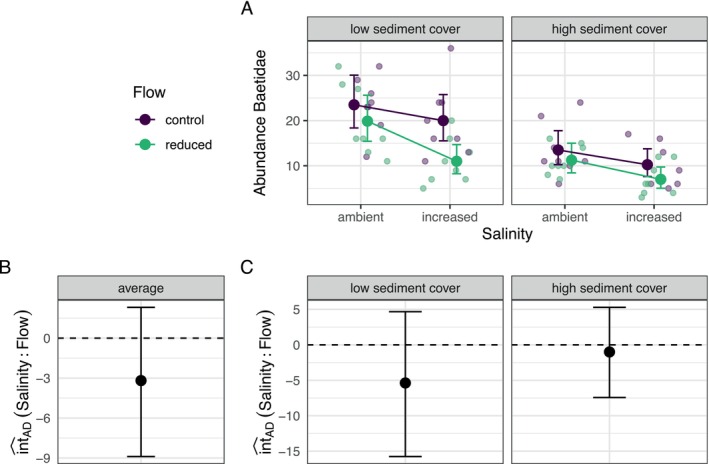
Example interaction analysis for a combination of two factorial stressors. (A) Baetid abundance given three anthropogenic stressors (flow velocity, salinity and fine sediment). Observations are given as transparent points. Adjusted predictions from the regression model (negative binomial GLM) are given as solid points. (B) Average estimate for the interaction between salinity and flow velocity for the simple addition null model. Error bars indicate 95% confidence intervals. (C) Estimates for the interaction between salinity and flow velocity for the simple addition null model in each of the fine sediment treatments. Data from Beermann et al. (2018).

Here, β_0_ to β_7_ are the estimated model coefficients, *Abund* is baetid abundance, and *Sa*, *Se*, and *Fl* refer to the three stressors, salinity, fine sediment, and flow velocity. Baetid abundances were negatively affected by all three stressors (fine sediment: χ^2^_1_ = 30.867, *p* < 0.001, flow velocity: χ^2^_1_ = 10.829, *p* < 0.001, salinity: χ^2^_1_ = 13.844, *p* < 0.001; Figure 5A). To test the AD instead of the MU (imposed by the logarithmic link function), we used post‐estimation inference and treated fine sediment as a covariate. We calculated the average ${\hat{\mathrm{int}}}_{\mathrm{AD}}$ between salinity and flow velocity across observations and separately for both fine sediment treatments. On average, the joint effect of the two stressors did not deviate from the AD (Figure 5B). This was also observed within each fine sediment treatment separately (Figure 5C). Therefore, the data do not provide sufficient evidence to reject the null hypothesis that the stressors combine additively.

### 4.2 Case Study 2: Gradient–Factor Interaction

In a lake mesocosm experiment, Greco et al. (2023) investigated the effect of increasing chloride concentrations under ambient (mesotrophic, 13.6 μg/L phosphorus and 330 μg/L nitrogen, *N* = 30) and high (meso‐eutrophic, 31.4 μg/L phosphorus and 1297 μg/L nitrogen, *N* = 30) nutrient conditions on planktonic communities. Ten days after nutrient addition, they added zooplankton, phytoplankton, and protists at densities comparable to those in the adjacent lake (Long Lake, Ontario, Canada) to the mesocosms. At the same time, they also initiated the salinity treatment by establishing a gradient ranging from ambient chloride concentrations (0.41 mg/L) to 1500 mg/L. Phytoplankton and protists were predominantly determined to the genus level and counted from water samples taken 6 weeks after starting the salinity treatment (Greco et al. 2023). Here, we use DO as a null model to investigate if the negative effect of high chloride concentrations on phytoplankton/protist richness is mitigated by higher nutrient availability.

We fitted a Poisson GAM with a logarithmic link function of the form:

(11) $$\ln\left(\text{Rich}.\mathrm{pp}\right)=\beta_{0}+\beta_{1}\mathrm{nut}+f\left(\text{chloride}\right)+f\left(\text{chloride},\mathrm{nut}\right)$$

Nutrient conditions (*nut*) were specified as a factorial variable (ambient vs. high). Chloride concentration was included as a smoothing term to capture nonlinear relationships, and a difference smoother was used to capture potential interactions between the two stressors. We used thin‐plate regression splines as the smoothing basis and the Restricted Maximum Likelihood (REML) criterion for smoothness selection. For post‐estimation inference with the DO, we relied on the unconditional variance–covariance matrix of the regression model to quantify uncertainty of the interaction estimate (see Data S2). Confidence intervals based on the unconditional matrix take the uncertainty of smoothness selection into account (Wood 2006). The unconditional confidence intervals tend to over‐cover (Wood 2006), i.e., the interval is more likely to cover the true value for an estimate than the nominal coverage probability suggests. Therefore, it becomes less probable to falsely conclude that an interaction is present.

**FIGURE 6:**
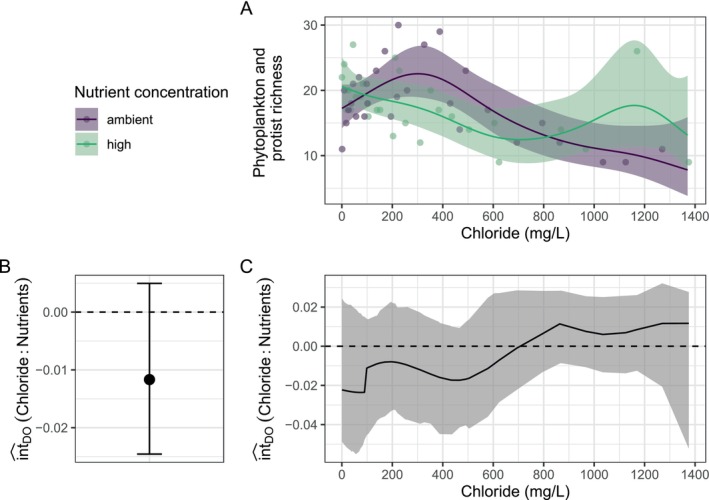
Example interaction analysis for a combination of a factorial stressor (nutrient concentration) and a stressor gradient (chloride concentration). (A) Phytoplankton and protist richness along a chloride gradient for ambient and high nutrient concentrations. Adjusted predictions from the regression model (Poisson GAM) are given as a solid line, while observations are given as transparent points. (B) Average estimate for the two‐stressor interaction for the dominance null model. (C) Estimates for the two‐stressor interaction for the dominance null model across the chloride gradient. Error bars and shaded areas indicate 95% confidence intervals. Data from Greco et al. (2023).

Chloride negatively affected phytoplankton and protist richness (χ^2^_4.1_ = 24.33, *p* < 0.001), while nutrient increase did not show a main effect (χ^2^_1_ = 0.1, *p* = 0.747). For ambient nutrient concentrations, there was an initial increase in richness up to 300 mg/L of chloride, followed by a steep decrease (Figure 6A). For high nutrient concentrations, richness decreased for low to medium chloride concentrations and showed a small increase between approx. 700 and 1200 mg/L chloride. However, this increase was driven by a single observation and is therefore more likely to reflect an outlier than a true pattern. Overall, chloride tended to have a more negative effect under high nutrient conditions; however, the deviation from the DO was nonsignificant (Figure 6B) and inconsistent in the direction across the chloride gradient (Figure 6C). Please note that the confidence intervals across the gradient are pointwise, and we would need to correct for multiple testing if we wanted to interpret pointwise deviations from the DO. From our analysis, we conclude that nutrient addition did not mitigate the negative effect of chloride on phytoplankton and protist richness.

### 4.3 Case Study 3: Gradient–Gradient Interaction

Raby et al. (2019) conducted 96‐h toxicity tests for four freshwater invertebrates with single and binary mixtures of the insecticide imidacloprid and the fungicide tebuconazole. Here, we focus on the joint effects of the toxicants on the survival rate of the amphipod *Hyalella azteca* . We aim to answer the question of whether the two toxicants in combination produce a higher mortality than expected from the MU. Null model choice was based on the assumption that the toxicants have different modes of action and that sensitivities are uncorrelated. Raby et al. (2019) first conducted single‐toxicant lethal concentration tests to determine the toxicant dose at which 50% of the individuals die (LC50). This allows translating toxicant doses to toxic units (TU), i.e., the toxicant concentration divided by its LC50 value. The mixture tests were then done with five fixed dose ratios (1:0, 0.67:0.33, 0.5:0.5, 0.33:0.67, 0:1) for six total toxicity levels (ΣTU: 0.125, 0.25, 0.5, 1, 2, 4) and a negative control. Each of the combinations was tested in two replicates with 10 individuals of *Hyalella azteca* each (Raby et al. 2019), resulting in a total of 620 individuals tested.

After identifying nonlinear patterns, we fitted a binomial GAM with a logit link function of the form:

(12) $$\text{logit}\left(\text{survival}\right)=\beta_{0}+f\left(\mathrm{IMI}\right)+f\left(\mathrm{TBZ}\right)+f\left(\mathrm{IMI},\mathrm{TBZ}\right)$$

Imidacloprid (IMI) and tebuconazole (TBZ) were given as measured concentrations of each toxicant, and the response variable was survival rate. We used thin‐plate regression splines for the single smoothers and cubic regression splines for the interaction smoother.

**FIGURE 7:**
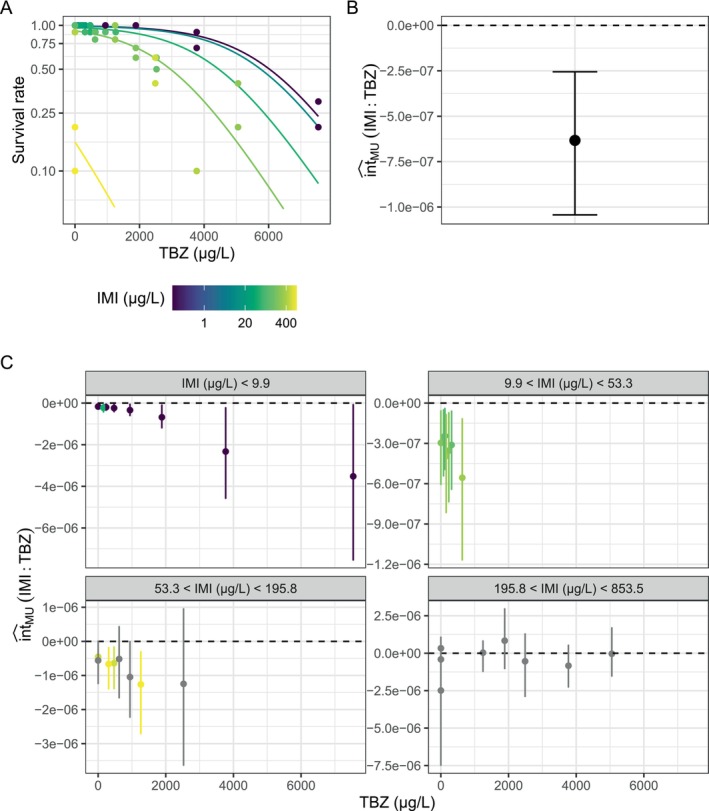
Example interaction analysis for a combination of two toxicant gradients (IMI, imidacloprid; TBZ, tebuconazole). (A) Survival rate of *Hyalella azteca* in dependence of TBZ and IMI doses. IMI doses are indicated by the color gradient. Adjusted predictions from the regression model (binomial GAM) are given as a solid line, and observations as points. Nonparallel lines indicate deviation from the multiplicative null model, because the y‐axis is log‐transformed. (B) Average estimate for the two‐stressor interaction for the multiplicative null model. (C) Observation‐wise estimates for the two‐stressor interaction given the multiplicative null model. Coloration is gray, if confidence intervals include 0, or indicates the IMI dose for those points along the toxicant gradients where the interaction is statistically significant. Error bars indicate 95% confidence intervals. Data from Raby et al. (2019).

Both toxicants strongly reduced the survival of *H. azteca* (imidacloprid: χ^2^_5.6_ = 89.86, *p* < 0.001, tebuconazole: χ^2^_1_ = 98.49, *p* < 0.001, Figure 7A). With increasing doses of imidacloprid, the relative change in survival caused by a particular dose of tebuconazole became stronger (Figure 7A). Therefore, we found a synergistic interaction between the two toxicants relative to the MU (Figure 7B). This interaction can consistently be detected for low to intermediate doses of imidacloprid but becomes nonsignificant for higher doses (Figure 7C).

## 5 Type I Error Rate Based on Case Studies

We evaluated type I error rate—the probability of incorrectly rejecting the tested null model—based on 10,000 simulated data sets for each null model across the three empirical case studies. Data simulations were performed as follows: First, we fitted the regression models described above for the case studies. From these regression models, we quantified the single‐stressor effects as a basis to calculate null‐model predictions for the AD, MU, and DO. The predictions served as reference values for simulations, utilizing the regression model's distributional assumptions (factor–factor interaction case study: negative binomial distribution with an estimated dispersion parameter of 11.98, factor–gradient interaction: Poisson distribution, gradient–gradient interaction: binomial distribution with 10 individuals per experimental unit). To each of the simulated data sets, we fitted the same regression model structure used for the empirical dataset and quantified the frequency of statistically significant average interaction estimates calculated for the true null model. The full R script for data simulations is provided in our OSF data repository (https://osf.io/J4DBZ/).

For the factor–factor interaction, type I error rate was close to 5% for all null models (Table 1). When stressor gradients were investigated using GAMs, type I error rate deviated from the expected 5%. The AD and DO had slightly inflated type I error rates for the factor–gradient interaction (6.85% and 7.61%), and lower type I error rates for the gradient–gradient interaction (0.82% and 0.84%). This shows that the confidence intervals calculated for post‐estimation inference should be interpreted with care. Nevertheless, even the highest type I error rate observed in our case studies (7.61%) was still within an acceptable range. For GAMs, there are a variety of options for model specification and a more extensive simulation study would be necessary to investigate how these choices influence performance of the confidence intervals for the interaction estimates. For example, we used a difference smoother to represent the factor–gradient interaction for case study 2, and we used a tensor interaction smoother for the gradient–gradient interaction in case study 3. Alternative formulations of these GAMs and different smoothing bases than the ones specified in our examples could be used, instead, and may affect type I error rate of post‐estimation inference. Therefore, we recommend accompanying the analysis with an evaluation of type I error rate using data simulations. For this purpose, the R script we provide in our data repository (https://osf.io/J4DBZ/) can be adapted for other datasets and regression models.

**TABLE 1:** Type I error rate for the average interaction estimate of the true null model. Single‐stressor effects were extracted from the regression model fitted to empirical data and combined according to each of the null models. These null model predictions were used to simulate 10,000 datasets for which average interaction estimates were calculated.

	Simple addition	Multiplicative	Dominance
Factor–factor interaction	5.32%	5.87%	5.12%
Factor–gradient interaction	6.85%	4.21%	7.61%
Gradient–gradient interaction	0.82%	5.33%	0.84%

## 6 Prospects and Limitations

The calculation of interaction estimates and their uncertainty from fitted regression models offers a flexible, yet complex analytical approach. We argue, however, that this complexity is necessary to advance multiple‐stressor research. In medical and socio‐political research, this approach facilitates the interpretation of study results by presenting them on an intuitive scale, the natural scale of the response, instead of relying on interaction coefficients that operate on a nonlinear scale (e.g., Ai and Norton 2003; McCabe et al. 2022; Mize 2019; VanderWeele and Knol 2014). We also advocate incorporating post‐estimation inference into ecological research. In ecology, nonlinear stressor–response relationships are a common phenomenon. To make ecological experiments more informative, there is a call to shift the experimental design toward stressor gradients instead of factorial stressors (Collins et al. 2022; Kreyling et al. 2018; Orr et al. 2020; Thomas and Ranjan 2024). Stressor interactions may also often be nonlinear (Duncan and Kefford 2021) and require more nuanced analyses than a simple classification as synergistic, antagonistic, or additive (Kefford et al. 2023). With the approach described here, the analyst can choose a specific multiple‐stressor null model and explore how stressor interactions change across gradients to differentiate, for example, between regions of synergism and regions where no interaction can be observed (see Figure 7C). Such detailed interpretation of ecological data can contribute to the refinement and development of hypotheses about the mechanisms of stressor interactions.

It is also important to recognize the distinction between a statistical interaction, i.e., a statistically significant deviation from null model predictions, and an intrinsic (“true”) stressor interaction (sensu Didham et al. 2007; Schäfer et al. 2023). Intrinsic interactions refer to the mechanistic pathway of how stressors jointly affect a biological response. Broadly, Schäfer et al. (2023) distinguish between *intensity interactions*, which involve the direct modification of one stressor's intensity by a second stressor, and *effect interactions*, where the co‐occurrence of two or more stressors results in the modification of each other's effect on the biological response variable without any numerical changes in stressor intensity. As an example of intensity interactions, intensive agricultural practices involve high water demands and can reduce base flow and current velocity (Hendriks et al. 2014) while also increasing fine sediment loads in streams through soil erosion (Ator 2019). The reduced current velocity increases sediment deposition, thereby intensifying the local accumulation of fine sediment. This can increase the net effect of the stressor combination if fine sediment is the proximate cause for the focal biological response (e.g., Beermann et al. 2018; Blöcher et al. 2020; Juvigny‐Khenafou et al. 2021b). In contrast, effect interactions can, for example, be caused by metabolic trade‐offs or altered biotic interactions (Beauchesne et al. 2021; Thompson et al. 2018a). Acclimation to pollutants requires metabolically expensive detoxification, and any additional metabolic stress from temperature, food deficiency, or oxygen limitation can increase the individual's sensitivity to the pollutant (Dinh et al. 2022; Liess et al. 2016; Moe et al. 2013; Zandalinas et al. 2021).

Statistical interactions can reflect these intrinsic interactions. However, they can also arise from incorrect assumptions: Null model choice might have been misguided, the statistical model might be incorrectly specified, or the ecological scale the stressors operate on could be different from that which is measured (Orr et al. 2024). This potential scale discrepancy is important to consider when interpreting mechanistic pathways (Boyd and Brown 2015; Simmons et al. 2021). Often, ecological studies focus on responses that are measured at the population‐ or community‐level (e.g., abundances and species richness), whereas stressors can be assumed to target individuals and their physiology (Ashauer and Jager 2018). These individual‐level effects drive changes in responses at higher organizational levels, such as populations, communities, or ecosystem functions (Kroeker et al. 2017; Simmons et al. 2021). Thompson et al. (2018b) have demonstrated that stressor effects that combine multiplicatively at the population level do not follow the MU at the community level. They therefore propose a compositional null model that accurately aggregates population‐level responses. However, this approach still requires the selection of a null model for the population‐level net effects of stressors, and it can introduce additional uncertainty caused by the aggregation process (Orr et al. 2021). Alternatively, we can incorporate ecological theories such as resource uptake theory or trophic networks into process‐based modeling frameworks to predict how stressor effects propagate from lower to higher organizational levels (Beauchesne et al. 2021; De Laender 2018; Simmons et al. 2021). Nevertheless, empirically informed stressor–response functions form the basis for predictions of cumulative effects when combined with causal effect pathways that link the most proximate cause for ecological change to the ultimate stressor (Jarvis et al. 2024). Understanding how stressors combine at the start of these causal effect pathways is crucial for predicting multiple‐stressor effects, even at higher organizational levels, and testing for stressor interactions remains an important task. However, when the causal effect pathways leading to a community‐ or ecosystem‐level response are poorly understood, testing both the AD and MU might be a good option because they provide complementary information (Spake et al. 2023). While the AD reflects absolute changes which are crucial for management decisions in conservation and relate to redistribution processes, the MU reflects relative changes which more accurately represent the multiplicative processes of population growth.

Here, we have focused on two‐way interactions based on the three null models of the co‐tolerance framework. However, higher‐order interactions may often play an important role in ecological change (Diamant et al. 2023), and more complex null models from ecotoxicological research may also be useful for environmental stressor combinations (Schäfer et al. 2023). For example, Kefford et al. (2023) investigated how two common anthropogenic stressors affected family richness of sensitive aquatic insect orders (Ephemeroptera, Plecoptera and Trichoptera, EPT) in riffle and edge habitats of Australian streams. They implicitly used the MU as their multiple‐stressor null model and found that the presence and direction of interactive effects between salinization and turbidity were dependent on habitat, terrain slope, and temperature (Kefford et al. 2023). Our approach can also be extended for identifying three‐way interactions; nevertheless, we argue that multiple‐stressor experiments should first focus on a mechanistic understanding of two‐way interactions before addressing more complex scenarios. Similarly, the concept of post‐estimation inference can also be extended to other multiple‐stressor null models, such as the concentration addition or stress addition null model. This would require a mathematical definition of interaction estimates specific to the respective null model.

## 7 Conclusions

Changing the scale of a response variable either through data transformations or link functions in regression models changes the null assumption for stressor combinations (e.g., Duncan and Kefford 2021; Spake et al. 2023). However, until now, there is no guidance in the ecological literature on how to test specific hypotheses on multiple‐stressor combinations if interaction terms in the statistical model do not immediately relate to these hypotheses. Here, we have presented mathematical definitions of null‐model‐specific interaction estimates and an approach to evaluate their statistical significance based on a variety of regression models. By using post‐estimation inference, researchers can (1) select a null model a priori based on theory and the addressed research question, (2) specify the statistical model that is most appropriate for the empirical data, (3) test the selected null model statistically, and (4) discuss mechanisms that can explain potential deviations from the null model. This has the potential to advance hypothesis‐driven research on stressor interactions by equipping the analyst with the statistical tools needed to flexibly test a specified null model.

## Author Contributions

**Iris Madge Pimentel:** conceptualization (lead), data curation (equal), formal analysis (lead), methodology (lead), software (lead), visualization (lead), writing – original draft (lead), writing – review and editing (equal). **Dania Albini:** conceptualization (supporting), writing – review and editing (equal). **Arne J. Beermann:** data curation (equal), writing – review and editing (equal). **Florian Leese:** funding acquisition (lead), writing – review and editing (equal). **Samuel J. Macaulay:** conceptualization (supporting), writing – review and editing (equal). **Christoph D. Matthaei:** writing – review and editing (equal). **James A. Orr:** conceptualization (supporting), data curation (equal), methodology (supporting), validation (equal), writing – review and editing (equal). **Jeremy J. Piggott:** writing – review and editing (equal). **Ralf B. Schäfer:** conceptualization (supporting), methodology (supporting), supervision (lead), validation (equal), writing – review and editing (equal).

## Conflicts of Interest

The authors declare no conflicts of interest.

## Supporting information


**Data S1:** ece371959‐sup‐0001‐Supinfo01.docx.

**Data S2:** ece371959‐sup‐0002‐Supinfo02.docx.

## Data Availability

The case study data sets and R scripts are publicly available at osf.io: DOI 10.17605/OSF.IO/J4DBZ (https://osf.io/J4DBZ/).
