# Supplementary material for: Hypothesis‐Driven Research on Multiple Stressors: An Analytical Framework for Stressor Interactions
Source: Ecol Evol. 2025 Aug 12;15(8):e71959. doi: 10.1002/ece3.71959 (PMC12340609; doi:10.1002/ece3.71959)
Supplement: Supplementary file 1 — Data S1: ece371959‐sup‐0001‐Supinfo01.docx. [file ECE3-15-e71959-s002.docx]

# Supplementary Material 1 for “Hypothesis-Driven Research on Multiple Stressors: An Analytical Framework for Stressor Interactions”

**Authors**: Iris Madge Pimentel^1^, Dania Albini^2^, Arne J. Beermann^1,3^, Samuel J. Macaulay^4^, Florian Leese^1,3^, Christoph D. Matthaei^5^, James A. Orr^6^, Jeremy J. Piggott^7^, Ralf B. Schäfer^8,3^

1 Aquatic Ecosystem Research, University of Duisburg-Essen, Essen, Germany

2 School of Life Sciences, University of Essex, Wivenhoe Park, United Kingdom

3 Centre for Water and Environmental Research (ZWU), University of Duisburg-Essen, Essen, Germany

4 Department of Biology, University of Oxford, Oxford, United Kingdom

5 Department of Zoology, University of Otago, Dunedin, New Zealand

6 School of the Environment, University of Queensland, Brisbane, Australia

7 Discipline of Zoology and Trinity Centre for the Environment, Trinity College Dublin, Dublin 2, Ireland

8 Research Centre One Health Ruhr and Faculty of Biology, Ecotoxicology, University of Duisburg-Essen, Essen, Germany

## Supplementary Material 1.1: Redefinition of the dominance null model

In previous definitions of the dominance null model (DO), the biological response under joint stressor exposure was predicted to take the lowest value observed among each of the single-stressor exposures (Schäfer and Piggott, 2018), or the value that deviates strongest from control conditions (Morris et al., 2022). However, these definitions do not consistently align with positively correlated sensitivities according to the co-tolerance concept (see Vinebrooke et al., 2004). We illustrate this by re-labelling control and stressor treatments in a hypothetical experiment. In our hypothetical experiment, stressor A and stressor B are combined in low and high intensity conditions. The choice of what is labelled as “control treatment” and what is labelled as “stressor treatment” is arbitrary, e.g., high and low temperature, or high and low nutrient concentrations can both be stressful for certain organisms. Let us assume that low intensity conditions for both stressors are optimal, and all 20 species of the initial community persist under these optimal conditions. Let us also assume that species’ sensitivities to higher intensities are positively correlated, and that all 6 species that are lost for high intensity conditions of stressor A are a subset of the 10 species that are lost for high intensity conditions of stressor B. Therefore, the number of species after the experiment is 14, 10 and 10, for the combination of high and low, low and high, or high and high intensity of stressor A and stressor B, respectively.

Table S1: Labelling options for stressor and control conditions. Stressor A and stressor B are factorial and combined in low or high intensity conditions, respectively. In this hypothetical experiment, however, the choice of control condition is arbitrary, and we can label any combination of low or high stressor intensities as experimental control.

| Stressor A intensity | Stressor B intensity | Observed species richness | Treatment label options | | | |
| --- | --- | --- | --- | --- | --- | --- |
|  |  |  | 1 | 2 | 3 | 4 |
| Low | Low | 20 | C | S_A_ | S_B_ | S_A,B_ |
| High | Low | 14 | S_A_ | C | S_A,B_ | S_B_ |
| Low | High | 10 | S_B_ | S_A,B_ | C | S_A_ |
| High | High | 10 | S_A,B_ | S_B_ | S_A_ | C |

Theoretically, we have four options how to label our control treatment (Table S1). Desirably, this choice should not affect the alignment of the predictions of the DO with our experimental observations. However, both previous mathematical definitions of the DO are sensitive to the treatment labels (Table S2). Therefore, we have formulated a new definition that is consistent with positively correlated sensitivities, independent of treatment labels (Table S2).

Usually, the choice of treatment labels is not completely arbitrary. Control conditions are given by the environmental conditions the organisms were taken from, or the conditions they were exposed to during an acclimatization phase. Nevertheless, the argument above is important as soon as the experimental manipulation, which would be labelled as stressor treatment, results in a higher response, e.g., in higher species richness, than the control condition does.

Table S2: Alignment of definitions for the dominance null model (DO) with positively correlated sensitivities according to the co-tolerance concept. For the two previous definitions of the DO, the alignment depends on the labelling of stressor treatments (see Table S1). Only our definition consistently aligns with positively correlated sensitivities. Misalignments are marked by the non-equal sign; alignments are highlighted in bold letters. *In Schäfer & Piggott (2017), the null models are defined to predict mortality instead of survival. The mortality for the joint stressor treatment is predicted to take the maximum value of the two single-stressor treatments, which can be re-formulated for survival (or species richness instead of lost species) as specified in the table.

| Definition of DO | Treatment label option | (Mis-)Alignment with co-tolerance concept |
| --- | --- | --- |
| Schäfer & Piggott (2017)*:  $S_{12}=C-max \left( C-S_{1}, {C-S}_{2} \right)$ | 1 | $\mathbf{10}=\mathbf{20}-\mathbf{max} \left( \mathbf{6}, \mathbf{10} \right)$ |
|  | 2 | $\mathbf{10}=\mathbf{14}-\mathbf{max} \left( -\mathbf{6}, \mathbf{4} \right)$ |
|  | 3 | $14\neq10-max \left( 0, -10 \right)$ |
|  | 4 | $20\neq10-max \left( 0, -4 \right)$ |
| Morris et al. (2021):  $S_{12}=\left\{ \begin{aligned} S_{1}, \left\vert C-S_{1} \right\vert>\left\vert{C-S}_{2} \right\vert\\ S_{2}, otherwise \end{aligned} \right.$ | 1 | $\mathbf{10}=\mathbf{10}$ $(\left\vert\mathbf{6} \right\vert<\left\vert\mathbf{10} \right\vert)$ |
|  | 2 | $10\neq20 (\left\vert-6 \right\vert>\left\vert4 \right\vert)$ |
|  | 3 | $14\neq20$ $(\left\vert0 \right\vert<\left\vert-10 \right\vert)$ |
|  | 4 | $20\neq14$ $(\left\vert0 \right\vert<\left\vert-4 \right\vert)$ |
| Our definition:  $\min\left( {C, S}_{12} \right)=\min\left( S_{1}, S_{2} \right)$ | 1 | $\min\left( \mathbf{20},\mathbf{10} \right)=\min\left( \mathbf{14}, \mathbf{10} \right)$ |
|  | 2 | $\min\left( \mathbf{14},\mathbf{10} \right)=\min\left( \mathbf{20}, \mathbf{10} \right)$ |
|  | 3 | $\min\left( \mathbf{10},\mathbf{14} \right)=\min\left( \mathbf{10}, \mathbf{20} \right)$ |
|  | 4 | $\min\left( \mathbf{10},\mathbf{20} \right)=\min\left( \mathbf{10}, \mathbf{14} \right)$ |

## Supplementary Material 1.2: Null-model predictions can become inaccurate if the response metric is continuous

The co-tolerance framework can best be applied to discrete response variables such as abundances or species richness. For continuous responses such as biomass, we first need to make assumptions about how stressors combine within individuals: If a specimen is affected by both focal stressors — i.e., it shows a reduction in biomass — do the stressor effects add up, do their relative effects multiply, does the strongest effect dominate, or do they even combine in a different way? For binary responses at the individual level (e.g., survival or death), the outcome for joint stressor exposure for individuals negatively affected by both stressors would be death, and there is no other way how the stressors could combine.

Even if we assume that stressor effects follow the same null model at the individual and population level, we can expect that continuous responses have different effect sizes among individuals (i.e., the same stressor can cause different biomass changes in two individuals). This can cause population-level predictions from the dominance and multiplicative null model to diverge from those derived from aggregating null-model predictions at the individual level (Table S3). Even if we assume that sensitive individuals respond to both stressors by the same absolute (Table S4) or relative change (Table S5) in biomass, the multiplicative null model can still result in inaccurate predictions at the population level, if individual contributions to total biomass differ. While the deviations in our examples are minor, they could become much stronger depending on how different individual contributions to total biomass and individual responses to stressors are. These examples should only demonstrate why it is conceptually difficult to extend the co-tolerance concept to continuous responses.

Table S3: If the response variable is continuous and multiple-stressor effects differ within and between individuals, population-level predictions from the multiplicative and dominance null model will be inaccurate. Individual (and total) biomass given single-stressor exposure are indicated within the same column above each other. Biomass change through single-stressor exposures is indicated in bold for the first stressor and in italics for the second stressor. Calculation of null-model predictions for the simple addition (AD), multiplicative (MU) and dominance (DO) null model are based on the equations in the main document. If these predictions are accurate, they are highlighted in bold, while those deviating from aggregated individual responses are highlighted in italics.

| Individual | Biomass in mg | Biomass in mg, negative sensitivity correlation | | | Biomass in mg, no sensitivity correlation | | Biomass in mg, positive sensitivity correlation | |
| --- | --- | --- | --- | --- | --- | --- | --- | --- |
|  | Control | Single stressors | Combined stressors | | Single stressors | Combined stressors | Single stressors | Combined stressors |
| Ind. A | 10 | **8** | | 8 | **8** | 8 | **8** | 8 |
|  |  | 10 | |  | 10 |  | 10 |  |
| Ind. B | 10 | **6** | | 6 | **6** | 6 | **6** | 6 |
|  |  | 10 | |  | 10 |  | *8* |  |
| Ind. C | 10 | **8** | | 8 | **8** | 6.4 | **8** | 6 |
|  |  | 10 | |  | *8* |  | *6* |  |
| Ind. D | 10 | 10 | | 8 | 10 | 6 | 10 | 10 |
|  |  | *8* | |  | *6* |  | 10 |  |
| Ind. E | 10 | 10 | | 6 | 10 | 10 | 10 | 10 |
|  |  | *6* | |  | 10 |  | 10 |  |
| Ind. F | 10 | 10 | | 10 | 10 | 10 | 10 | 10 |
|  |  | 10 | |  | 10 |  | 10 |  |
| Total biomass | 60 | 52 | | 46 | 52 | 46.4 | 52 | 50 |
|  |  | 54 | |  | 54 |  | 54 |  |
|  |  | Prediction (AD):  **46** | | | Prediction (MU):  *46.8* | | Prediction (DO):  *52* | |

Table S4: If the response variable is continuous, population-level predictions from the multiplicative null model can be inaccurate, even if absolute stressor effects are identical between individuals and stressors (-3 mg). Individual (and total) biomass given single-stressor exposure are indicated within the same column above each other. Biomass change through single-stressor exposures is indicated in bold for the first stressor and in italics for the second stressor. Calculation of null-model predictions for the simple addition (AD), multiplicative (MU) and dominance (DO) null model are based on the equations in the main document. If these predictions are accurate, they are highlighted in bold, while those deviating from aggregated individual responses are highlighted in italics.

| Individual | Biomass in mg | Biomass in mg, negative sensitivity correlation | | | Biomass in mg, no sensitivity correlation | | Biomass in mg, positive sensitivity correlation | |
| --- | --- | --- | --- | --- | --- | --- | --- | --- |
|  | Control | Single stressors | Combined stressors | | Single stressors | Combined stressors | Single stressors | Combined stressors |
| Ind. A | 9 | **6** | | 6 | **6** | 6 | **6** | 6 |
|  |  | 9 | |  | 9 |  | 9 |  |
| Ind. B | 15 | **12** | | 12 | **12** | 12 | **12** | 12 |
|  |  | 15 | |  | 15 |  | *12* |  |
| Ind. C | 12 | **9** | | 9 | **9** | 6.75 | **9** | 9 |
|  |  | 12 | |  | *9* |  | *9* |  |
| Ind. D | 12 | 12 | | 9 | 12 | 9 | 12 | 12 |
|  |  | *9* | |  | *9* |  | 12 |  |
| Ind. E | 12 | 12 | | 9 | 12 | 12 | 12 | 12 |
|  |  | *9* | |  | 12 |  | 12 |  |
| Ind. F | 9 | 9 | | 9 | 9 | 9 | 9 | 9 |
|  |  | 9 | |  | 9 |  | 9 |  |
| Total biomass | 69 | 60 | | 54 | 60 | 54.75 | 60 | 60 |
|  |  | 63 | |  | 63 |  | 63 |  |
|  |  | Prediction (AD):  **54** | | | Prediction (MU):  *54.78* | | Prediction (DO):  **60** | |

Table S5: If the response variable is continuous, population-level predictions from the multiplicative null model can be inaccurate, even if relative stressor effects are identical between individuals and stressors (×66.6%). Individual (and total) biomass given single-stressor exposure are indicated within the same column above each other. Biomass change through single-stressor exposures is indicated in bold for the first stressor and in italics for the second stressor. Calculation of null-model predictions for the simple addition (AD), multiplicative (MU) and dominance (DO) null model are based on the equations in the main document. If these predictions are accurate, they are highlighted in bold, while those deviating from aggregated individual responses are highlighted in italics.

| Individual | Biomass in mg | Biomass in mg, negative sensitivity correlation | | | Biomass in mg, no sensitivity correlation | | Biomass in mg, positive sensitivity correlation | |
| --- | --- | --- | --- | --- | --- | --- | --- | --- |
|  | Control | Single stressors | Combined stressors | | Single stressors | Combined stressors | Single stressors | Combined stressors |
| Ind. A | 9 | **6** | | 6 | **6** | 6 | **6** | 6 |
|  |  | 9 | |  | 9 |  | 9 |  |
| Ind. B | 15 | **10** | | 10 | **10** | 10 | **10** | 10 |
|  |  | 15 | |  | 15 |  | *10* |  |
| Ind. C | 12 | **8** | | 8 | **8** | 5.33 | **8** | 8 |
|  |  | 12 | |  | *8* |  | *8* |  |
| Ind. D | 12 | 12 | | 6 | 12 | 8 | 12 | 12 |
|  |  | *6* | |  | *8* |  | 12 |  |
| Ind. E | 12 | 12 | | 6 | 12 | 12 | 12 | 12 |
|  |  | *6* | |  | 12 |  | 12 |  |
| Ind. F | 9 | 9 | | 9 | 9 | 9 | 9 | 9 |
|  |  | 9 | |  | 9 |  | 9 |  |
| Total biomass | 69 | 57 | | 45 | 57 | 50.33 | 57 | 57 |
|  |  | 57 | |  | 61 |  | 60 |  |
|  |  | Prediction (AD):  **45** | | | Prediction (MU):  *50.39* | | Prediction (DO):  **57** | |

## Supplementary Material 1.3: Mathematical definition of interaction estimates

Here, we show how we derived interaction estimate definitions for each null model based on the equations predicting joint stressor exposure. In each equation, *C, S_A_, S_B_* and *S_A,B_* denote the biological response for control conditions, stressor conditions A and B, and for joint stressor exposure. This biological response can be estimated from a fitted regression model and the elements of each equation can be replaced by these regression model estimations. Let $f\left( a,b | x \right)$ be the conditional function that describes the regression model’s estimations in dependence on the two focal stressors *a* and *b,* while all other covariates are kept constant at values specified in the vector $x$. Moreover, *c_A_* and *c_B_* describe the factor levels (or values for a gradient) that are considered control conditions for stressor a and b, respectively, and *s_A_* and *s_B_* refer to the stressor conditions. Then it follows:

$$C=f\left( c_{A},c_{B} | x \right)$$

$$S_{A}=f\left( s_{A},c_{B} | x \right)$$

$$S_{B}=f\left( c_{A},s_{B} | x \right)$$

$$S_{A,B}=f\left( s_{A},s_{B} | x \right)$$

This replacement will be used for all interaction estimate definitions below.

**Simple addition null model**

$$S_{A,B}\boldsymbol{=}S_{A}+S_{B}-C$$

$$0\boldsymbol{=}{S_{A}+S_{B}-C-S}_{A,B}$$

$$0=S_{A,B}-S_{A}-S_{B}+C$$

$$0=f\left( s_{A},s_{B} | x \right)-f\left( s_{A},c_{B} | x \right)-f\left( c_{A},s_{B} | x \right)+f\left( c_{A},c_{B} | x \right)$$

Now, we can use the right-hand side of the equation to define an interaction estimate that takes the value 0 if stressor effects combine according to the simple addition null model (AD). To standardize the interaction estimate by the amount of change in the two focal stressors, we first divide by the product of the differences in stressor intensity between each stressor and control conditions.

$$0=\frac{f\left( s_{A},s_{B} | x \right)-f\left( s_{A},c_{B} | x \right)-f\left( c_{A},s_{B} | x \right)+f\left( c_{A},c_{B} | x \right)}{\left( s_{A}-c_{A} \right)\times\left( s_{B}-c_{B} \right)}$$

$$\hat{int}_{AD}:=\frac{f\left( s_{A},s_{B} | x \right)-f\left( s_{A},c_{B} | x \right)-f\left( c_{A},s_{B} | x \right)+f\left( c_{A},c_{B} | x \right)}{\left( s_{A}-c_{A} \right)\times\left( s_{B}-c_{B} \right)}$$

**Multiplicative null model**

$$S_{A,B}\boldsymbol{=}\frac{S_{A}\times S_{B}}{C}$$

$$1\boldsymbol{=}\frac{S_{A}\times S_{B}}{C\times S_{A,B}}$$

Because we aimed to define an interaction estimate that takes the value 0 if stressor effects combine according to the multiplicative null model (MU), we take the natural logarithm for both sides of the equation.

$$0=\ln\left( \frac{S_{A}\times S_{B}}{C\times S_{A,B}} \right)$$

$$0={ln(S}_{A})+\ln\left( S_{B} \right)-\ln\left( C \right)-ln(S_{A,B})$$

$$0=\ln\left( S_{A,B} \right)-\ln\left( S_{A} \right)-\ln\left( S_{B} \right)+\ln\left( C \right)$$

$$0=\ln\left[ f\left( s_{A},s_{B} | x \right) \right]-\ln\left[ f\left( s_{A},c_{B} | x \right) \right]-\ln\left[ f\left( c_{A},s_{B} | x \right) \right]+\ln\left[ f\left( c_{A},c_{B} | x \right) \right]$$

In parallel to the definition for the interaction estimate of the AD above, we standardize by the amount of change in stressor intensities.

$$0=\frac{\ln\left[ f\left( s_{A},s_{B} | x \right) \right]-\ln\left[ f\left( s_{A},c_{B} | x \right) \right]-\ln\left[ f\left( c_{A},s_{B} | x \right) \right]+\ln\left[ f\left( c_{A},c_{B} | x \right) \right]}{\left( s_{A}-c_{A} \right)\times\left( s_{B}-c_{B} \right)}$$

$$\hat{int}_{MU}:=\frac{\ln\left[ f\left( s_{A},s_{B} | x \right) \right]-\ln\left[ f\left( s_{A},c_{B} | x \right) \right]-\ln\left[ f\left( c_{A},s_{B} | x \right) \right]+\ln\left[ f\left( c_{A},c_{B} | x \right) \right]}{\left( s_{A}-c_{A} \right)\times\left( s_{B}-c_{B} \right)}$$

**Dominance null model**

$$\min\left( C, S_{A,B} \right)\boldsymbol{=}\min\left( S_{A}, S_{B} \right)$$

$$0\boldsymbol{=}\min\left( S_{A}, S_{B} \right)-\min\left( C, S_{A,B} \right)$$

$$0\boldsymbol{=}\min\left( C, S_{A,B} \right)-\min\left( S_{A}, S_{B} \right)$$

$$0\boldsymbol{=}\min\left[ f\left( c_{A},c_{B} | x \right), f\left( s_{A},s_{B} | x \right) \right]-\min\left[ f\left( s_{A},c_{B} | x \right), f\left( c_{A},s_{B} | x \right) \right]$$

In parallel to the definitions for the interaction estimate of the AD and MU above, we standardize by the amount of change in stressor intensities.

$$0=\frac{\min\left( f\left( c_{A},c_{B} | x \right), f\left( s_{A},s_{B} | x \right) \right)-\min\left( f\left( s_{A},c_{B} | x \right), f\left( c_{A},s_{B} | x \right) \right)}{\left( s_{A}-c_{A} \right)\times\left( s_{B}-c_{B} \right)}$$

$$\hat{int}_{DO}:=\frac{\min\left( f\left( c_{A},c_{B} | x \right), f\left( s_{A},s_{B} | x \right) \right)-\min\left( f\left( s_{A},c_{B} | x \right), f\left( c_{A},s_{B} | x \right) \right)}{\left( s_{A}-c_{A} \right)\times\left( s_{B}-c_{B} \right)}$$
