# Supplementary material for: Hypothesis‐Driven Research on Multiple Stressors: An Analytical Framework for Stressor Interactions
Source: Ecol Evol. 2025 Aug 12;15(8):e71959. doi: 10.1002/ece3.71959 (PMC12340609; doi:10.1002/ece3.71959)
Supplement: Supplementary file 2 — Data S2: ece371959‐sup‐0002‐Supinfo02.docx. [file ECE3-15-e71959-s001.docx]

Supplementary material 2 for: “Hypothesis-Driven Research on Multiple Stressors: An Analytical Framework for Stressor Interactions”. Case studies

**Authors**: Iris Madge Pimentel^1^, Dania Albini^2^, Arne J. Beermann^1,3^, Samuel J. Macaulay^4^, Florian Leese^1,3^, Christoph D. Matthaei^5^, James A. Orr^6^, Jeremy J. Piggott^7^, Ralf B. Schäfer^8,3^

1 Aquatic Ecosystem Research, University of Duisburg-Essen, Essen, Germany

2 School of Life Sciences, University of Essex, Wivenhoe Park, United Kingdom

3 Centre for Water and Environmental Research (ZWU), University of Duisburg-Essen, Essen, Germany

4 Department of Biology, University of Oxford, Oxford, United Kingdom

5 Department of Zoology, University of Otago, Dunedin, New Zealand

6 School of the Environment, University of Queensland, Brisbane, Australia

7 Discipline of Zoology and Trinity Centre for the Environment, Trinity College Dublin, Dublin 2, Ireland

8 Research Centre One Health Ruhr and Faculty of Biology, Ecotoxicology, University of Duisburg-Essen, Essen, Germany

Iris Madge Pimentel

2025-03-07

# Introduction and setup

In this document, we present an annotated version of the R code used to analyze three empirical data sets from published studies for stressor interactions. We test for deviations from three multiple-stressor null models: From the simple addition (AD), multiplicative (MU) and dominance (DO) null model. We fit generalized linear models (GLMs) or generalized additive models (GAMs) and calculate interaction estimates for a specific null model from the regression model’s adjusted predictions. Uncertainty estimation for statistical inference is done with the Krinsky-and-Robb method.

The following R packages are required: *tidyverse* for data handling, *mgcv* to fit GAMs, *DHARMa* for residual diagnostics of regression models, and *marginaleffects* for post-estimation hypothesis testing. Moreover, we use *viridis* for color scales in *ggplot()*.

library(tidyverse)
library(mgcv)
library(DHARMa)
library(marginaleffects)
library(viridis)

set.seed(1) # for reproducibility

We load in the example data sets used as case studies. One data set will be used to exemplify an interaction analysis for two factorial stressors, one for a factorial-gradient combination, and the last data set combines two stressor gradients.

df.factorial <- read_csv("data/factorial.csv")

## Rows: 64 Columns: 6
## ── Column specification ────────────────────────────────────────────────────────
## Delimiter: ","
## chr (3): Salinity, Sediment, Flow
## dbl (3): Mesocosm, Baetidae.benthic, Baetidae.leaf
##
## ℹ Use `spec()` to retrieve the full column specification for this data.
## ℹ Specify the column types or set `show_col_types = FALSE` to quiet this message.

df.mixed <- read_csv("data/mixed.csv")

## Rows: 58 Columns: 4
## ── Column specification ────────────────────────────────────────────────────────
## Delimiter: ","
## chr (1): nut
## dbl (3): Mesocosm, chloride, Rich.pp
##
## ℹ Use `spec()` to retrieve the full column specification for this data.
## ℹ Specify the column types or set `show_col_types = FALSE` to quiet this message.

df.gradient <- read_csv("data/gradient.csv")

## Rows: 310 Columns: 8
## ── Column specification ────────────────────────────────────────────────────────
## Delimiter: ","
## chr (1): Species
## dbl (7): IMI, TBZ, total, survival, IMI_TU, TBZ_TU, TU_Sum
##
## ℹ Use `spec()` to retrieve the full column specification for this data.
## ℹ Specify the column types or set `show_col_types = FALSE` to quiet this message.

For post-estimation inference, we use two custom-made functions throughout the case studies. The first function (*nd.generator()*) is used to prepare a ‘counterfactual’ data frame. The resulting data frame has four replicates of the original data set, and a column “dataset” that specifies the identity of these replicates. One replicate represents control conditions (dataset: “C”), the second replicate represents an increase in the first focal stressor (“S1”), the third an increase in the other stressor (“S2”) and the fourth represent a simultaneous increase in both stressors (“S12”). We have to provide the control-condition data set, and specify the desired increase in stressor intensity for gradients.

We recommend to provide a control dataset (*df.ref*) which has any focal factorial stressor set to the reference level and any focal continuous stressor at its original value (see example case studies below). The increase in stressor intensity could be set to 0.01% of the gradient length.

#' Generate data frame for hypothesis testing
#'
#' Creates a data frame with four replicates of a reference dataset, which in
#' itself represents control conditions. The first replicate is the reference
#' dataset itself (C), the second represents single-stressor conditions for
#' stressor 1 (S1), the third represents single-stressor conditions for stressor
#' 2 (S2) and the last represents joint stressor exposure (S12).
#'
#' @param df.ref Data frame of reference conditions (i.e., control scenario) at
#' which a multiple-stressor null model should be evaluated.
#' @param stressor1 Name of the first stressor in your data frame (character).
#' @param stressor2 Name of the second stressor in your data frame (character).
#' @param diff Either NULL (for factorial stressors), a single numeric
#' (for one numeric stressor), or a numeric vector of length 2 (for two numeric
#' stressors). Specifies how much to increase each stressor.
#' @return A data frame with 4 modified datasets representing control, S1, S2,
#' and S12 stressor scenarios.
#' @examples
#' nd.generator(control_df, "Salinity", "Flow", diff = NULL)
#' nd.generator(control_df, "nut", "chloride", diff = 10)
#'
#' @export
nd.generator <- function(df.ref, stressor1, stressor2, diff = NULL){
 df.prep <- df.ref |>
 dplyr::mutate(dataset = "C", rowidcf = dplyr::row_number())

 if (is.null(diff)) {
 df.out <- df.prep |>
 tibble::add_case(
 df.prep |> dplyr::mutate(
 !!stressor1 := factor(levels(.data[[stressor1]])[
 as.numeric(.data[[stressor1]]) + 1],
 levels = levels(.data[[stressor1]])
 ),
 dataset = "S1"
 )
 ) |>
 tibble::add_case(
 df.prep |> dplyr::mutate(
 !!stressor2 := factor(levels(.data[[stressor2]])[
 as.numeric(.data[[stressor2]]) + 1],
 levels = levels(.data[[stressor2]])
 ),
 dataset = "S2"
 )
 ) |>
 tibble::add_case(
 df.prep |> dplyr::mutate(
 !!stressor1 := factor(levels(.data[[stressor1]])[
 as.numeric(.data[[stressor1]]) + 1],
 levels = levels(.data[[stressor1]])
 ),
 !!stressor2 := factor(levels(.data[[stressor2]])[
 as.numeric(.data[[stressor2]]) + 1],
 levels = levels(.data[[stressor2]])
 ),
 dataset = "S12"
 )
 )
 } else if (length(diff) == 1) {
 if (is.factor(df.prep[[stressor1]])) {
 df.out <- df.prep |>
 tibble::add_case(df.prep |> dplyr::mutate(
 !!stressor1 := factor(levels(.data[[stressor1]])[
 as.numeric(.data[[stressor1]]) + 1],
 levels = levels(.data[[stressor1]])
 ),
 dataset = "S1"
 )) |>
 tibble::add_case(df.prep |> dplyr::mutate(
 !!stressor2 := .data[[stressor2]] + diff,
 dataset = "S2"
 )) |>
 tibble::add_case(df.prep |> dplyr::mutate(
 !!stressor1 := factor(levels(.data[[stressor1]])[
 as.numeric(.data[[stressor1]]) + 1],
 levels = levels(.data[[stressor1]])
 ),
 !!stressor2 := .data[[stressor2]] + diff,
 dataset = "S12"
 ))
 } else {
 df.out <- df.prep |>
 tibble::add_case(df.prep |> dplyr::mutate(
 !!stressor1 := .data[[stressor1]] + diff,
 dataset = "S1"
 )) |>
 tibble::add_case(df.prep |> dplyr::mutate(
 !!stressor2 := factor(levels(.data[[stressor2]])[
 as.numeric(.data[[stressor2]]) + 1],
 levels = levels(.data[[stressor2]])
 ),
 dataset = "S2"
 )) |>
 tibble::add_case(df.prep |> dplyr::mutate(
 !!stressor1 := .data[[stressor1]] + diff,
 !!stressor2 := factor(levels(.data[[stressor2]])[
 as.numeric(.data[[stressor2]]) + 1],
 levels = levels(.data[[stressor2]])
 ),
 dataset = "S12"
 ))
 }
 } else if (length(diff) == 2) {
 df.out <- df.prep |>
 tibble::add_case(dplyr::mutate(df.prep,
 !!stressor1 := .data[[stressor1]] + diff[1],
 dataset = "S1")) |>
 tibble::add_case(dplyr::mutate(df.prep,
 !!stressor2 := .data[[stressor2]] + diff[2],
 dataset = "S2")) |>
 tibble::add_case(dplyr::mutate(df.prep,
 !!stressor1 := .data[[stressor1]] + diff[1],
 !!stressor2 := .data[[stressor2]] + diff[2],
 dataset = "S12"))
 }

 if ("rowid" %in% names(df.out)) {
 df.out <- dplyr::select(df.out, -rowid)
 }

 return(df.out)
}

The second function tests for stressor interactions given each of the three multiple-stressor null models treated here and needs to be used in combination with the *predictions()* function from the *marginaleffects* package. The data frame produced with *nd.generator()* will be provided to this function.

#' Calculate stressor interactions given a multiple-stressor null model
#'
#' Given model predictions on counterfactual datasets generated with the
#' # nd.generator() function, this function estimates interaction effects
#' relative to one of three null models from the co-tolerance framework:
#' simple addition (AD), multiplicative (MU), or dominance (DO).
#'
#' @param df.cf A data frame containing counterfactual predictions with:
#' - `rowidcf`: Row identifier for matched predictions.
#' - `dataset`: One of "C", "S1", "S2", or "S12".
#' - `estimate`: Model-adjusted predictions on the response scale.
#' @param stressor1 Character. Name of the first stressor.
#' @param stressor2 Character. Name of the second stressor.
#' @param strategy Character. Either `"rowwise"` or `"average"`. Rowwise will
#' return an interaction esitmate for each row of the reference/control dataset,
#' while average will average all interaction estimates (either across all rows,
#' or by a another variable specifies in by)
#' @param by Optional character. Grouping variable for averaging.
#' @param null Character. One of `"ad"`, `"mu"`, `"do"`, or `"all"`.
#'
#' @return A tibble of interaction estimates.
#' @export
hypothesis.function <- function(df.cf, stressor1, stressor2,
 strategy = c("average", "rowwise"),
 by = NULL,
 null = c("ad", "mu", "do", "all")) {
 strategy <- match.arg(strategy)
 null <- match.arg(null)

 df <- df.cf |>
 dplyr::arrange(dataset, rowidcf)

 C.df <- dplyr::filter(df, dataset == "C")
 S1.df <- dplyr::filter(df, dataset == "S1")
 S2.df <- dplyr::filter(df, dataset == "S2")
 S12.df <- dplyr::filter(df, dataset == "S12")

 change_x1 <- as.numeric(S12.df[[stressor1]]) - as.numeric(C.df[[stressor1]])
 change_x2 <- as.numeric(S12.df[[stressor2]]) - as.numeric(C.df[[stressor2]])

 C <- C.df$estimate
 S1 <- S1.df$estimate
 S2 <- S2.df$estimate
 S12 <- S12.df$estimate

 if (null == "ad") {
 row.est <- (S12 - S1 - S2 + C) / (change_x1 * change_x2)
 } else if (null == "mu") {
 row.est <- (log(S12) - log(S1) - log(S2) + log(C)) / (change_x1 * change_x2)
 } else if (null == "do") {
 row.est <- (pmin(S12, C) - pmin(S1, S2)) / (change_x1 * change_x2)
 }

 if (null != "all") {
 rowwise <- C.df |>
 dplyr::select(-estimate, -dataset) |>
 dplyr::mutate(
 estimate = row.est,
 term = null,
 change_x1 = change_x1,
 change_x2 = change_x2,
 .before = 1
 )
 }

 if (null == "all") {
 row.est.ad <- (S12 - S1 - S2 + C) / (change_x1 * change_x2)
 row.est.mu <- (log(S12) - log(S1) - log(S2) + log(C)) / (change_x1 * change_x2)
 row.est.do <- (pmin(S12, C) - pmin(S1, S2)) / (change_x1 * change_x2)

 rowwise <- C.df |>
 dplyr::select(-estimate, -dataset) |>
 dplyr::mutate(
 ad = row.est.ad,
 mu = row.est.mu,
 do = row.est.do,
 change_x1 = change_x1,
 change_x2 = change_x2
 ) |>
 tidyr::pivot_longer(cols = c("ad", "mu", "do"),
 names_to = "term", values_to = "estimate")
 }

 if (strategy == "average") {
 grouping <- "term"
 if (!is.null(by)) grouping <- c(grouping, by)
 out <- rowwise |>
 dplyr::group_by(dplyr::across(all_of(grouping))) |>
 dplyr::summarise(estimate = mean(estimate), .groups = "drop")
 } else {
 out <- rowwise
 }

 return(out)
}

These two functions are also provided as a stand-alone R file in the Open Science Framework depository:

Finally, we specify a standard plotting theme for the *ggplot()* function.

theme_set(theme_bw())

# Case study 1: Factor-factor interaction

The first case study is taken from Beermann et al. (2018). They conducted a full-factorial stream mesocosm experiment and studied responses of the macroinvertebrate community to three common anthropogenic stressors (salinization, flow velocity reduction and sediment input). We focus on changes in total abundances of the mayfly family Baetidae caused by two focal stressors: salinization and reduced flow velocity. In their study, Beermann et al. (2018) separately analyzed individual abundances from the benthic habitat (within the sediment) and from a leaf litter bag deposited in the mesocosms. We first aggregate these counts to analyze total abundances.

head(df.factorial)

## # A tibble: 6 × 6
## Mesocosm Salinity Sediment Flow Baetidae.benthic Baetidae.leaf
## <dbl> <chr> <chr> <chr> <dbl> <dbl>
## 1 1 salt sediment normal flow 5 1
## 2 2 no salt sediment reduced flow 9 6
## 3 3 no salt no sediment reduced flow 14 2
## 4 4 salt sediment normal flow 6 0
## 5 5 no salt no sediment normal flow 10 2
## 6 6 salt no sediment normal flow 20 4

# calculate total abundance
df.factorial <- df.factorial |>
 mutate(Abund = Baetidae.benthic + Baetidae.leaf,
 .keep = "unused")

# create factors
df.factorial$Flow <- factor(df.factorial$Flow, levels = c("normal flow", "reduced flow"))
df.factorial$Salinity <- factor(df.factorial$Salinity, levels = c("no salt", "salt"))
df.factorial$Sediment <- factor(df.factorial$Sediment, levels = c("no sediment", "sediment"))

# deviance coding
contrasts(df.factorial$Flow) <- contr.sum(2)
contrasts(df.factorial$Salinity) <- contr.sum(2)
contrasts(df.factorial$Sediment) <- contr.sum(2)

Baetid mayflies are sensitive to salinization and to reductions in oxygen supply. The mechanisms through which the stressors act on the organisms are supposed to be different: While we expect salinization to affect osmotic regulation, flow velocity reduction limits oxygen availability. The different mechanisms may result in negatively correlated sensitivities. Therefore, we choose the simple addition null model as our multiple-stressor null model and we aim to answer the question if a reduction in flow velocity and an increase in salinity exert a more than additive effect. This would indicate a stressor interaction that cannot be explained with negatively correlated sensitivities.

## Regression model specification and validation

Next, we fit a Poisson regression model and check model assumptions with residual plots. In the *glm()* function, we can specify the assumed conditional distribution of our response variable (family = poisson) and we specify the regression equation (Abund ~ Sa * Se * Fl). By using ’*’, we do not only include the three-way-interaction, but also all lower-level interactions terms and the main terms in the model.

mod.fac1 <- glm(Abund ~ Salinity * Sediment * Flow,
 family = poisson(), data = df.factorial)

# residual plots with DHARMa package
simulationOutput <- simulateResiduals(fittedModel = mod.fac1, plot = F)
plot(simulationOutput)


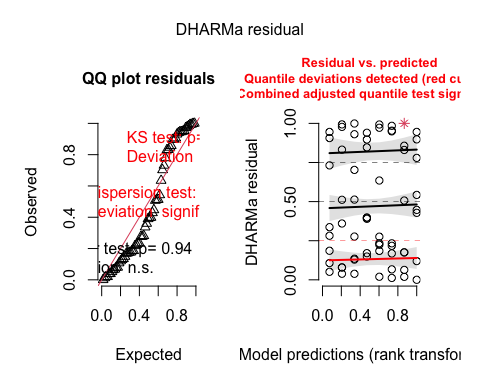


plotResiduals(simulationOutput, form = df.factorial$Salinity)


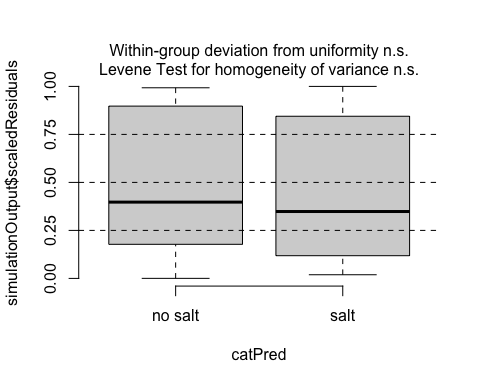


plotResiduals(simulationOutput, form = df.factorial$Sediment)


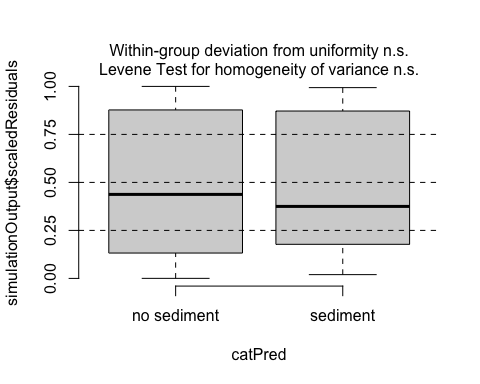


plotResiduals(simulationOutput, form = df.factorial$Flow)


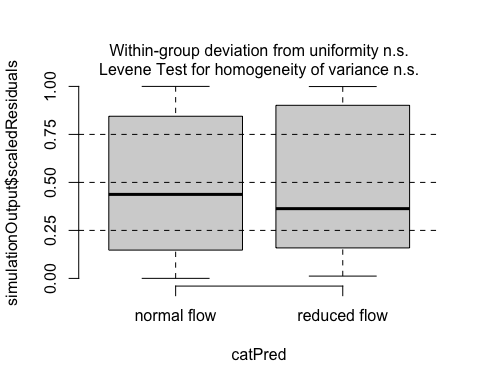


# dispersion test
testDispersion(mod.fac1, plot = F)

##
## DHARMa nonparametric dispersion test via sd of residuals fitted vs.
## simulated
##
## data: simulationOutput
## dispersion = 2.112, p-value < 2.2e-16
## alternative hypothesis: two.sided

The residual plots and dispersion test indicate overdispersion. This can be accounted for by using a negative binomial regression, instead. This can be specified in the *gam()* function (but not in *glm()*).

mod.fac2 <- gam(Abund ~ Salinity * Sediment * Flow,
 family = nb(), data = df.factorial)

# residual plots with DHARMa package
simulationOutput <- simulateResiduals(fittedModel = mod.fac2, plot = F)

## Registered S3 method overwritten by 'GGally':
## method from
## +.gg ggplot2

## Registered S3 method overwritten by 'mgcViz':
## method from
## +.gg GGally

plot(simulationOutput)


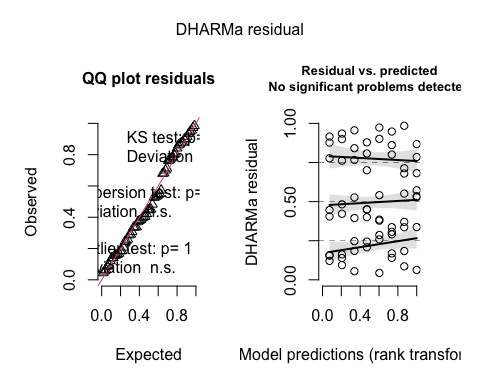


plotResiduals(simulationOutput, form = df.factorial$Salinity)


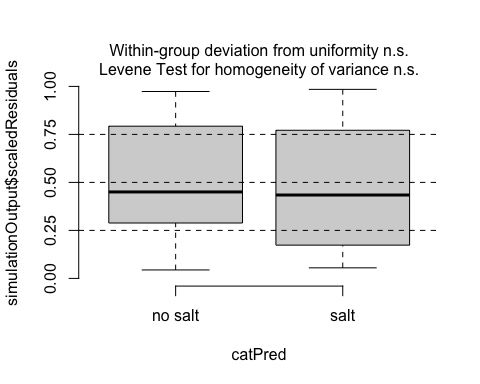


plotResiduals(simulationOutput, form = df.factorial$Sediment)


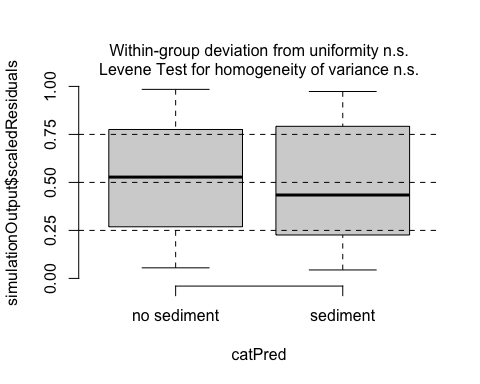


plotResiduals(simulationOutput, form = df.factorial$Flow)


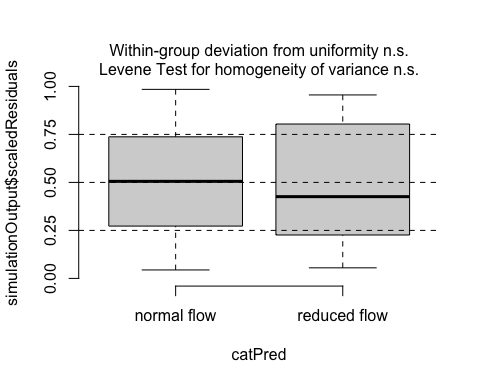


There are no obvious patterns in the residuals anymore and we can continue with model interpretation.

## Model interpretation: Plot model predictions

We can use the *anova()* command to obtain a statistical evaluation of the regression model’s main and interaction terms.

anova(mod.fac2)

##
## Family: Negative Binomial(11.98)
## Link function: log
##
## Formula:
## Abund ~ Salinity * Sediment * Flow
##
## Parametric Terms:
## df Chi.sq p-value
## Salinity 1 13.844 0.000199
## Sediment 1 30.867 2.76e-08
## Flow 1 10.829 0.000999
## Salinity:Sediment 1 0.000 0.994141
## Salinity:Flow 1 2.428 0.119170
## Sediment:Flow 1 0.249 0.617514
## Salinity:Sediment:Flow 1 0.328 0.566925

All three stressors affect baetid abundance. No interaction term was statistically significant, however, we cannot use the model output to evaluate the null model we want to test (AD). This is because we used a logarithmic link function. Therefore, the interaction terms of the regression model correspond to MU, but not to AD.

Before statistically analyzing the two-way interaction between flow velocity reduction and salinity, we plot the raw data and model predictions. This will also aid in the interpretation of any statistically significant interaction effects. To obtain appropriate confidence limits, we predict on the link scale (logarithmic) and then use the *exp()* function to calculate model predictions (and confidence limits) on the response scale.

pred.fac2 <- predictions(mod.fac2, type = "link") |>
 # translate to response scale
 mutate(estimate.resp = exp(estimate),
 conf.low.resp = exp(conf.low),
 conf.high.resp = exp(conf.high)) |>
 # reduce to unique observations
 select(Salinity, Sediment, Flow, estimate.resp, conf.low.resp, conf.high.resp) |>
 unique()

ggplot(data = pred.fac2, aes(x = Salinity, y = estimate.resp, color = Flow)) +
 geom_jitter(df.factorial, mapping = aes(y = Abund), alpha = 0.4, height = 0, width = 0.3) +
 geom_point(size = 3, position = position_dodge(width = 0.3)) +
 geom_errorbar(aes(ymin = conf.low.resp, ymax = conf.high.resp), width = 0.2,
 position = position_dodge(width = 0.3)) +
 geom_line(aes(group = Flow), position = position_dodge(width = 0.3)) +
 facet_wrap(facets = vars(Sediment)) +
 scale_color_viridis(discrete = T, begin = 0, end = 0.7) +
 labs(y = "Abundance Baetidae")


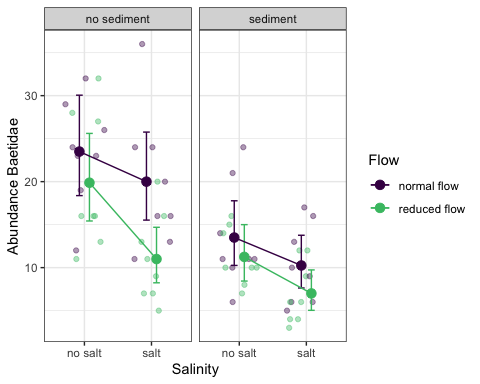


The addition of salt and the reduction in flow velocity reduce the abundance of Baetidae. If no sediment was added, the effect of reduced flow velocity seems to be more pronounced if the water was more saline. If sediment was added, the effect of reduced flow is similar for ambient and increased salt conditions.

## Post-estimation inference

We first focus on observation-wise two-way interaction estimates. For this, we prepare a data frame that has reference conditions only (ref.fac) and use our custom function to build the full data frame need for the hypothesis function (nd.fac).

ref.fac <- datagrid(model = mod.fac2,
 Salinity = "no salt",
 Flow = "normal flow",
 # counterfactual grid type sets all other covariates (e.g., sediment) to observed values
 grid_type = "counterfactual")

nd.fac <- nd.generator(ref.fac, stressor1 = "Salinity", stressor2 = "Flow")

Now, we can use our custom hypothesis function (*hypothesis.function()*) within the *predictions()* function and test the simple addition null model. The *inferences()* function is used to apply the K-R method for uncertainty estimation. Please note that this function is currently marked as ‘experimental’ in the *marginaleffects* package, and its functionality might be migrated to other functions in the future.

# simulations will take a while
hyp.fac <- predictions(mod.fac2,
 newdata = nd.fac,
 hypothesis = function(x) {
 hypothesis.function(x,
 null = "ad",
 stressor1 = "Salinity",
 stressor2 = "Flow",
 by = "Sediment")}) |>
 inferences(method = "simulation")

ggplot(data = hyp.fac, aes(x = term, y = estimate)) +
 geom_point(size = 3) +
 geom_errorbar(aes(ymin = conf.low, ymax = conf.high), width = 0.2) +
 facet_wrap(facets = vars(Sediment)) +
 labs(x = "Null model", y = "Interaction estimate (Salinity : Flow)") +
 theme(legend.position = "none") +
 geom_hline(yintercept = 0, linetype = "dashed")


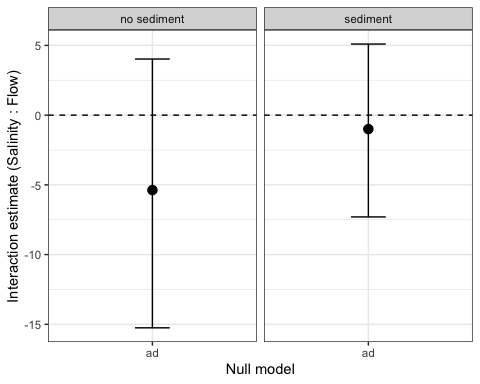


We have used the *by = “Sediment”* argument to average the interaction estimates for “no sediment” and “sediment” conditions separately. In fact, all interaction estimates for “no sediment” are identical to each other, and all interaction estimates for “sediment” are, too. This is because no other covariates are involved. The aggregation is simply done to end up with a table with two rows only instead of having a long table with two sets of 32 identical values.

For both sediment conditions, the interaction estimates for AD are statistically not significant. For inference across our sample, we rely on the overall average interaction estimate.

hyp.fac.avg <- predictions(mod.fac2,
 newdata = nd.fac,
 hypothesis = function(x) {
 hypothesis.function(x,
 null = "ad",
 stressor1 = "Salinity",
 stressor2 = "Flow",
 strategy = "average")}) |>
 inferences(method = "simulation")

ggplot(data = hyp.fac.avg, aes(x = term, y = estimate)) +
 geom_point(size = 3) +
 geom_errorbar(aes(ymin = conf.low, ymax = conf.high), width = 0.2) +
 labs(x = "Null model", y = "Interaction estimate (Salinity : Flow)") +
 theme(legend.position = "none") +
 geom_hline(yintercept = 0, linetype = "dashed")


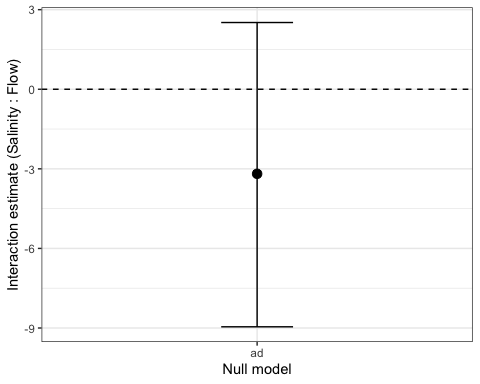


In conclusion, the data do not give sufficient evidence to reject AD as a null model.

# Case study 2: Factor-gradient interaction

This case study is based on Greco et al. (2023), who analysed the effect of increasing chloride concentrations (up to 1500 mg/L) on planktonic communities in lake mesocosms. The effect of chloride was analyzed under ambient nutrient conditions (A) and high nutrient conditions (H). We focus on phytoplankton and protist richness as a response variable.

The table contains information about (1) Mesocosm ID, (2) nutrient conditions (nut), (3) average chloride concentration over the course of the experiment (chloride, mg/L), and (4) phytoplankton and protist richness (Rich.pp).

head(df.mixed)

## # A tibble: 6 × 4
## Mesocosm nut chloride Rich.pp
## <dbl> <chr> <dbl> <dbl>
## 1 1 A 388 29
## 2 2 H 868. 14
## 3 3 A 1.09 11
## 4 4 H 152. 17
## 5 5 H 139 17
## 6 6 H 436 16

# remove rows without relevant data (1 row)
df.mixed <- df.mixed |> filter(!is.na(Rich.pp))

# create factor
df.mixed$nut <- factor(df.mixed$nut, levels = c("A", "H"),
 ordered = T)

We want to answer the question: Does the higher availability of nutrients given the high nutrient treatment ameliorate the negative effects of high chloride concentrations? Thus, we use DO as a null model, to investigate if phytoplankton/protist richness is different between ambient and high nutrient conditions when salinity is high.

## Model specification and validation

We fit a Poisson regression for taxon counts and check model assumptions with residual plots. We use the default options in *mgcv* with respect to smoothing splines: (Penalized) Thin plate regression splines are used with a basis dimension of 10. The interaction between chloride and nutrients is represented through the *by* argument within the second smoother. The interaction is set up by fitting a reference smoother for ambient conditions of nutrients and fitting a second smoother that represents the difference in richness between ambient and high nutrient conditions across the chloride gradient. For this, we need to specify nutrients as an ordered factor (see above). We use Restricted Maximum Likelihood (method = “REML”) for smoothing parameter estimation, because it is less prone to overfitting (Wood, 2011).

mod.mixed <- gam(Rich.pp ~ nut + s(chloride) + s(chloride, by = nut),
 data = df.mixed, family = poisson, method = "REML")

For model validation, we can still use the *DHARMa* pacakge. However, in addition, we should also check if the basis dimension of the smoother is sufficiently high.

# check smooth basis dimension
k.check(mod.mixed)

## k' edf k-index p-value
## s(chloride) 9 3.613275 1.148353 0.8375
## s(chloride):nutH 9 3.543449 1.148353 0.8575

# check residual plots with DHARMa
simulationOutput <- simulateResiduals(fittedModel = mod.mixed, plot = F)
plot(simulationOutput)


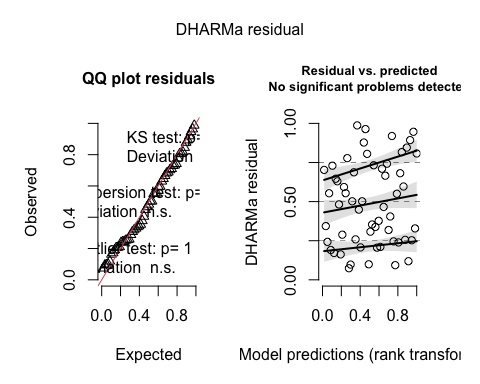


plotResiduals(simulationOutput, df.mixed$nut)


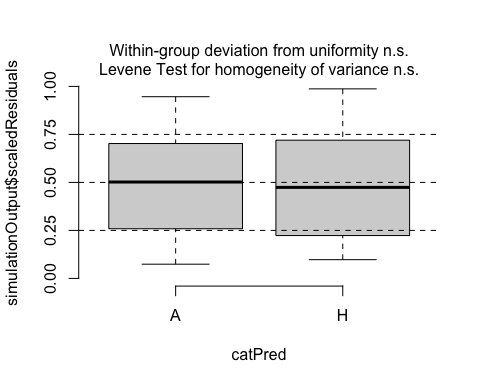


plotResiduals(simulationOutput, df.mixed$chloride)


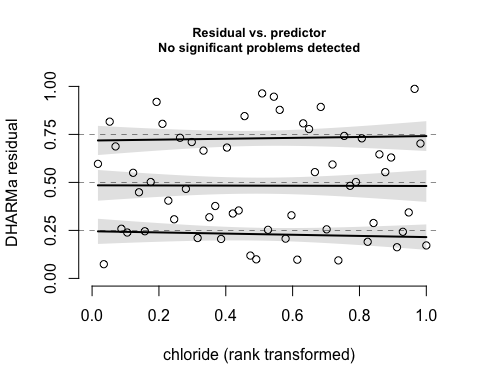


The basis dimension is sufficiently high and there are no strong patterns in the residual plots. We continue with model interpretation.

## Model interpretation and plotting model predictions

First, we take a look at the summary of our GAM.

anova(mod.mixed)

##
## Family: poisson
## Link function: log
##
## Formula:
## Rich.pp ~ nut + s(chloride) + s(chloride, by = nut)
##
## Parametric Terms:
## df Chi.sq p-value
## nut 1 0.104 0.747
##
## Approximate significance of smooth terms:
## edf Ref.df Chi.sq p-value
## s(chloride) 3.613 4.450 24.33 0.000144
## s(chloride):nutH 3.543 4.354 13.47 0.015862

There is no significant main effect of nutrients (given as a parametric coefficient). Chloride significantly affects pyhtoplankton/protist richness, however, we need to plot the smoothers to interpret this effect.

When constructing the point-wise confidence intervals around the model predictions, we use the unconditional variance-covariance matrix. This takes the additional uncertainty into account that is introduced by the selection of smoothing parameters. To produce a visually smooth chloride-response function, we create model predictions over a fine grid.

mod.mixed.pred <- predictions(mod.mixed,
 type = "link",
 # unconditional variance-covariance matrix
 vcov = vcov(mod.mixed, unconditional = T),
 # predict over fine grid to produce a smooth function
 newdata = datagrid(chloride = seq(min(df.mixed$chloride),
 max(df.mixed$chloride), 5),
 nut = unique)) |>
 # translate to response scale
 mutate(estimate.res = exp(estimate),
 conf.low.res = exp(conf.low),
 conf.high.res = exp(conf.high))

ggplot(data = mod.mixed.pred, aes(x = chloride, y = estimate.res, color = nut)) +
 geom_point(data = df.mixed, aes(y = Rich.pp), alpha = 0.4) +
 geom_ribbon(aes(ymin = conf.low.res, ymax = conf.high.res, y = estimate.res,
 fill = nut), color = NA,
 alpha = 0.3) +
 geom_line(aes(group = nut)) +
 scale_x_continuous(breaks = seq(0, 1500, 200)) +
 labs(x = "Chloride (mg/L)", y = "Phytoplankton and protist richness") +
 scale_color_viridis(discrete = T, begin = 0, end = 0.7) +
 scale_fill_viridis(discrete = T, begin = 0, end = 0.7)


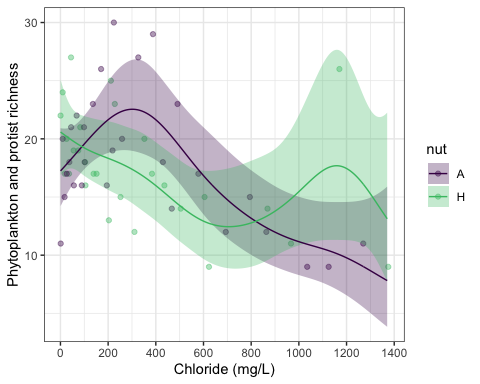


Generally, high chloride concentrations reduce protist and phytoplankton richness. For ambient nutrient conditions, however, richness peaks at low to moderate chloride concentrations (approx. 300 mg/L) and only decreases afterwards. For high nutrient availability, this peak is absent, but richness increases shortly between approx. 800 and 1200 mg/L. Thus, with high nutrient conditions, richness is predicted to be lower for low to moderate chloride concentrations, but higher for high chloride concentrations. We continue by analyzing the two-way interaction between the stressors in more detail.

## Post-estimation inference

We start by calculating an interaction estimate at every observation. For the chloride gradient, we approximate instantaneous change by calculating the change in the response variable for a small increase in chloride concentration. This small increase is 0.01% of the covered gradient. First, we prepare the reference grid, which consists of observed chloride values, and control conditions for nutrients. Then, we generate the data grid for single and joint stressor exposure, given the small increase in chloride concentrations (nd.mixed).

ref.mixed <- datagrid(model = mod.mixed,
 nut = "A",
 grid_type = "counterfactual")

# small value to add to chloride
DIFF <- range(df.mixed$chloride) |> diff() * 0.0001

nd.mixed <- nd.generator(ref.mixed,
 stressor1 = "nut",
 stressor2 = "chloride",
 diff = DIFF) |>
 mutate(nut = factor(nut, ordered = T))

Next, we estimate the row-wise interaction effects for DO and plot them along the gradient.

hyp.mixed <- predictions(mod.mixed,
 newdata = nd.mixed,
 vcov = vcov(mod.mixed, unconditional = T),
 type = "response",
 hypothesis = function(x){
 hypothesis.function(df.cf = x,
 null = "do",
 stressor1 = "nut",
 stressor2 = "chloride",
 strategy = "rowwise")}) |>
 inferences(method = "simulation")

ggplot(data = hyp.mixed, aes(x = chloride, y = estimate)) +
 geom_line() +
 geom_hline(yintercept = 0, linetype = "dashed") +
 geom_ribbon(aes(ymin = conf.low, ymax = conf.high), alpha = 0.2) +
 labs(x = "chloride", y = "Interaction estimate") +
 geom_rug(sides = "b")


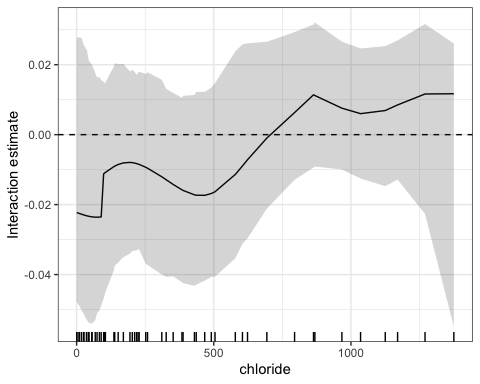


The confidence bands consistently include 0, giving no indication for an interaction according to the dominance null model. It is important to note that the confidence intervals that we constructed here are point-wise intervals. If we wanted to draw statistical conclusions on the interaction term across the function, we would need to construct simultaneous confidence bands (which would become wider). We will base our conclusion on a single aggregate measure: The average interaction estimate across our sample.

Before we do this, we want to draw attention to the sensitivity of our analysis to step-size for the approximation of slope.

diff.choice <- hyp.mixed |> mutate(DIFF = DIFF) |> tibble()

for(i in c(1:4, 6:10)){
 .DIFF <- range(df.mixed$chloride) |> diff() * 10^(-i)

 .nd.mixed <- nd.generator(ref.mixed,
 stressor1 = "nut",
 stressor2 = "chloride",
 diff = .DIFF) |>
 mutate(nut = factor(nut, ordered = T))

 .hyp.mixed <- predictions(mod.mixed,
 newdata = .nd.mixed,
 vcov = vcov(mod.mixed, unconditional = T),
 type = "response",
 hypothesis = function(x){
 hypothesis.function(df.cf = x,
 null = "do",
 stressor1 = "nut",
 stressor2 = "chloride",
 strategy = "rowwise")}) |>
 inferences(method = "simulation") |>
 mutate(DIFF = .DIFF)

 diff.choice <- add_case(diff.choice, .hyp.mixed)
 }

ggplot(data = diff.choice, aes(x = chloride, y = estimate)) +
 geom_line() +
 geom_hline(yintercept = 0, linetype = "dashed") +
 geom_ribbon(aes(ymin = conf.low, ymax = conf.high), alpha = 0.2) +
 facet_wrap(facets=vars(DIFF), scales = "free") +
 labs(x = "chloride", y = "Interaction estimate") +
 geom_rug(sides = "b")


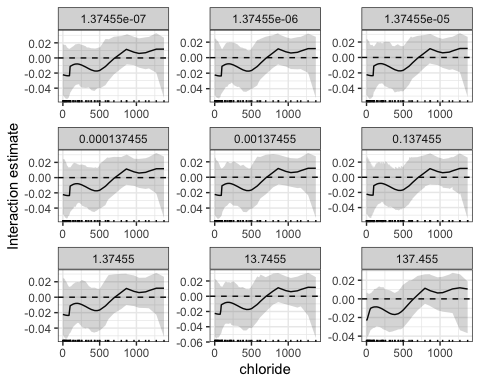


When approximating the slope, we should always check if the step size shows large differences in interpretation when changing it for smaller or larger values. In the current example, there is little effect of the step size (from 10% to 0.00000001% of the range). Only for unreasonably large steps (10%) the pattern seems to change slightly.

We use the originally chosen step size (0.01% of the gradient) and calculate the average interaction estimate for DO.

hyp.mixed.avg <- predictions(mod.mixed,
 newdata = nd.mixed,
 vcov = vcov(mod.mixed, unconditional = T),
 hypothesis = function(x){
 hypothesis.function(df.cf = x,
 null = "do",
 stressor1 = "nut",
 stressor2 = "chloride",
 strategy = "average")}) |>
 inferences(method = "simulation")

hyp.mixed.avg |> hypotheses(equivalence = c(-2,2)) |> tibble()

## # A tibble: 1 × 13
## term estimate std.error statistic p.value s.value conf.low conf.high
## <chr> <dbl> <dbl> <dbl> <dbl> <dbl> <dbl> <dbl>
## 1 do -0.0117 0.00594 -1.97 0.0494 4.34 -0.0233 -0.0000331
## # ℹ 5 more variables: statistic.noninf <dbl>, statistic.nonsup <dbl>,
## # p.value.noninf <dbl>, p.value.nonsup <dbl>, p.value.equiv <dbl>

ggplot(hyp.mixed.avg, aes(x = term, y = estimate)) + geom_point(size = 3) +
 geom_errorbar(aes(ymin = conf.low, ymax = conf.high), width = 0.2) +
 facet_wrap(facets = vars(term), scales = "free") +
 geom_hline(aes(yintercept = 0), linetype = "dashed") +
 labs(x = "null model", y = "interaction estimate (chloride : nutrients)")


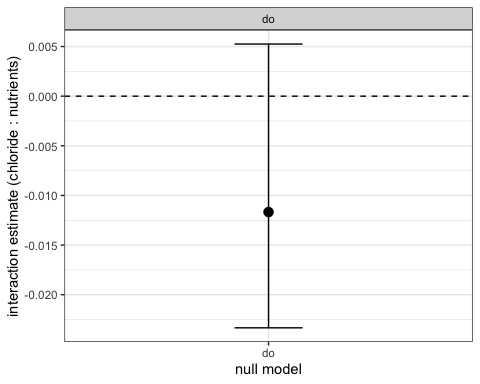


On average, the interaction between chloride and nutrient concentrations is non-significant for the dominance null model.

# Case study 3: Gradient-gradient interaction

The third case-study is based on Raby et al. (2019). They exposed four freshwater invertebrates to single and binary mixtures of an insecticide (imidacloprid) and a fungicide (tebuconazole, TBZ) using acute 96-hours toxicity tests. We focus on the response of the amphipod *Hyalella azteca* in our example analysis, and address the question, if the joint toxicity is more detrimental than expected according to MU as a null model.

First, we filter the original data set for the species of interest and calculate survival rate.

head(df.gradient)

## # A tibble: 6 × 8
## Species IMI TBZ total survival IMI_TU TBZ_TU TU_Sum
## <chr> <dbl> <dbl> <dbl> <dbl> <dbl> <dbl> <dbl>
## 1 Chironomus dilutus 0 0 10 10 0 0 0
## 2 Chironomus dilutus 0 0 10 10 0 0 0
## 3 Chironomus dilutus 0.9 0 10 10 0.1 0 0.1
## 4 Chironomus dilutus 0.9 0 10 10 0.1 0 0.1
## 5 Chironomus dilutus 1.9 0 10 8 0.3 0 0.3
## 6 Chironomus dilutus 1.9 0 10 9 0.3 0 0.3

df.gradient <- df.gradient |>
 filter(Species == "Hyalella azteca") |>
 mutate(survival_rate = survival/total)

The experimental design in this study does not fully combine the toxicant gradients. Instead, based on previous single-exposure toxicity tests, the authors used a ray design. Five toxic unit does ratios were chosen (1:0, 0.67:0.33, 0.5:0.5, 0.33:0.67, 0:1) and tested at 6 total dose levels. The design is visualized below.

ggplot(data = df.gradient, aes(x = IMI, y = TBZ)) +
 geom_point() +
 labs(x = "Imidacloprid (μg/L)", y = "Tebuconazole (μg/L)")


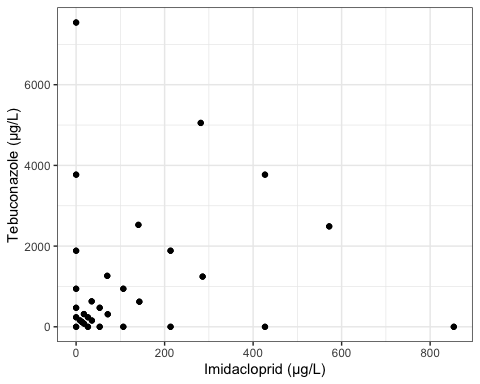


## Model specification and validation

We start by fitting a GLM to the data. We use binomial regression, because survival rate is expressed as proportion of individuals surviving. We indicate the total number of individuals in the “weights” argument.

mod.gradient1 <- glm(survival_rate ~ IMI * TBZ,
 data = df.gradient,
 family = binomial,
 weights = total)

# check residual plots with DHARMa
simulationOutput <- simulateResiduals(fittedModel = mod.gradient1, plot = F)

plot(simulationOutput)


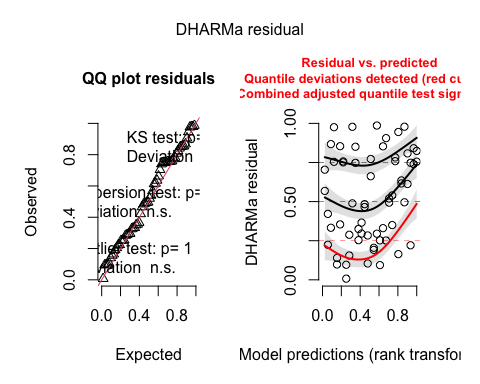


plotResiduals(simulationOutput, df.gradient$IMI)


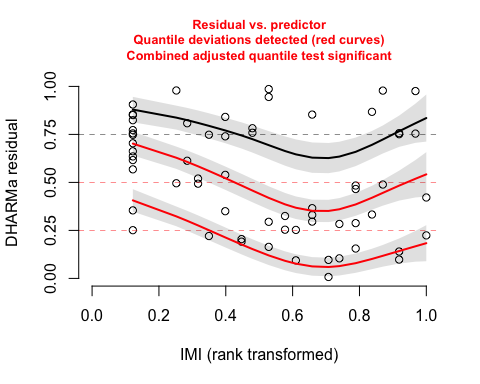


plotResiduals(simulationOutput, df.gradient$TBZ)


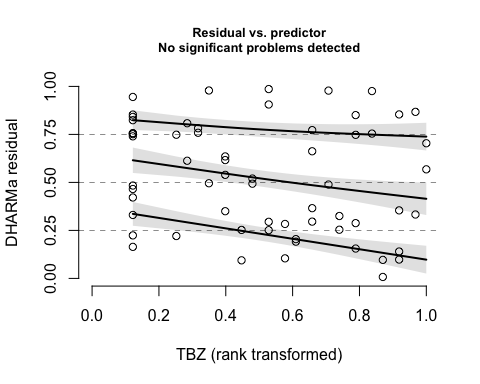


The patterns in the residuals indicate non-linearity. We use a GAM to account for this non-linearity. The *mgcv* package allows for an ANOVA-like decomposition into main effects (specified in *s()* below) and a tensor-product interaction (specified in *ti()* below). Again, we use the default options for the smoothing basis. By default, thin-plate splines are used in *s()*, and cubic regression splines are used in *ti()*.

mod.gradient <- gam(survival_rate ~ s(IMI) + s(TBZ) + ti(IMI, TBZ),
 weights = total,
 data = df.gradient,
 family = binomial,
 method = "REML")

# check basis dimensions
k.check(mod.gradient)

## k' edf k-index p-value
## s(IMI) 9 4.873608 0.9851367 0.5575
## s(TBZ) 9 1.000019 0.9565633 0.4575
## ti(IMI,TBZ) 16 1.000069 0.9797093 0.5300

# check residual plots with DHARMa
simulationOutput <- simulateResiduals(fittedModel = mod.gradient, plot = F)
plot(simulationOutput)


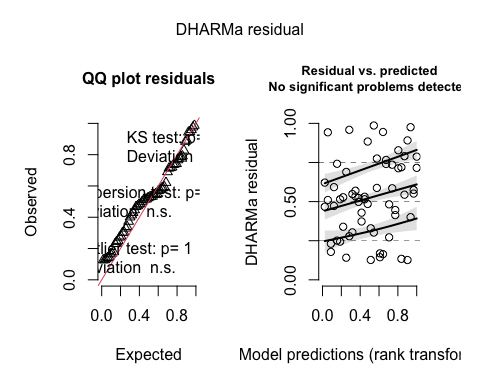


plotResiduals(simulationOutput, df.gradient$IMI)


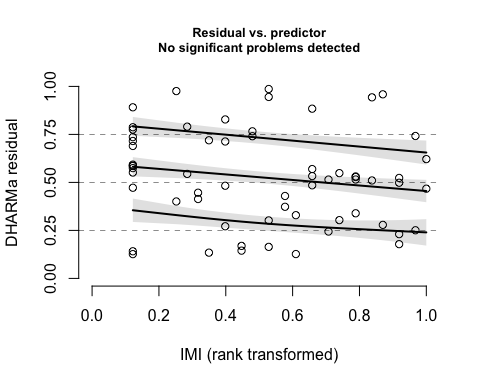


plotResiduals(simulationOutput, df.gradient$TBZ)


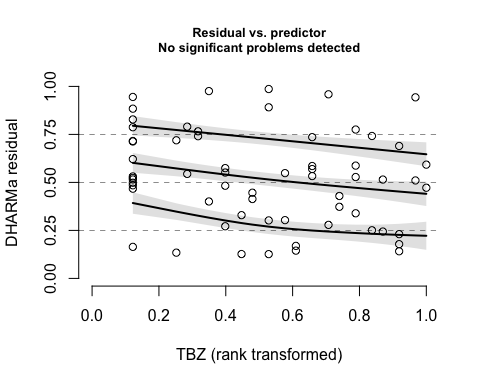


The residual plots have no strong patterns anymore. We continue with model interpretation.

## Model interpretation and plot model predictions

summary(mod.gradient)

##
## Family: binomial
## Link function: logit
##
## Formula:
## survival_rate ~ s(IMI) + s(TBZ) + ti(IMI, TBZ)
##
## Parametric coefficients:
## Estimate Std. Error z value Pr(>|z|)
## (Intercept) 2.3870 0.2034 11.74 <2e-16 ***
## ---
## Signif. codes: 0 '***' 0.001 '**' 0.01 '*' 0.05 '.' 0.1 ' ' 1
##
## Approximate significance of smooth terms:
## edf Ref.df Chi.sq p-value
## s(IMI) 4.874 5.684 90.860 <2e-16 ***
## s(TBZ) 1.000 1.000 98.486 <2e-16 ***
## ti(IMI,TBZ) 1.000 1.000 0.137 0.711
## ---
## Signif. codes: 0 '***' 0.001 '**' 0.01 '*' 0.05 '.' 0.1 ' ' 1
##
## R-sq.(adj) = 0.945 Deviance explained = 89.6%
## -REML = 62.666 Scale est. = 1 n = 62

We plot model prediction for a fine grid of TBZ concentrations at the minimum, lower quartile, median, upper quartile and maximum observed values of IMI (Tukey’s five numbers). The points indicate observations and the y-scale is log-transformed to aid in visual interpretation for MU as a null model.

mod.gradient.pred <- predictions(mod.gradient,
 newdata = datagrid(TBZ = seq(0, 7541.2, length.out = 50),
 IMI = fivenum),
 type = "link",
 # unconditional variance-covariance matrix
 vcov = vcov(mod.gradient, unconditional = T)) |>
 # translate to response scale: Here with plogis (inverse of logit-link)
 mutate(estimate.res = plogis(estimate),
 conf.low.res = plogis(conf.low),
 conf.high.res = plogis(conf.high))

ggplot(data = mod.gradient.pred, aes(x = TBZ, y = estimate.res,
 color = IMI + 0.01)) +
 geom_line(aes(group = IMI)) +
 labs(x = "Tebuconazole (μg/L)", y = "Survival rate") +
 geom_point(data = df.gradient, aes(y = survival_rate)) +
 scale_y_continuous(transform = "log10", limits = c(0.01, 1)) +
 scale_color_viridis(trans = 'log')

## Warning: Removed 29 rows containing missing values or values outside the scale range
## (`geom_line()`).


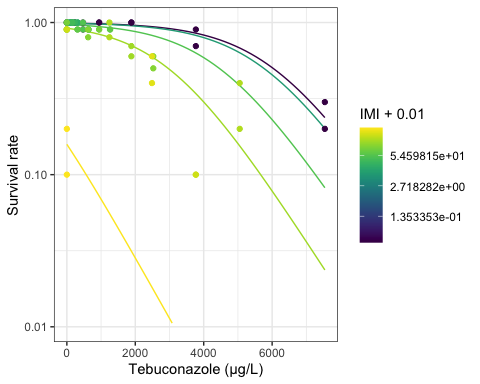


With higher IMI concentrations, the change in survival rate on the logarithmic scale becomes more negative for the same TBZ concentration. This would indicate a synergistic interaction with respect to MU.

## Post-estimation inference

We prepare the data grid for single and joint stressor exposure. For both toxicants, we choose a small increase of 0.01% of the covered gradient.

ref.gradient <- df.gradient

# small value to add to chloride
DIFF1 <- range(df.gradient$IMI) |> diff() * 0.0001
DIFF2 <- range(df.gradient$TBZ) |> diff() * 0.0001

nd.gradient <- nd.generator(ref.gradient,
 stressor1 = "IMI",
 stressor2 = "TBZ",
 diff = c(DIFF1, DIFF2))

Next, we estimate the row-wise interaction effects and plot it along the gradients. For an improved visualization, we facet the plots into first, second, thirs and fourth quartile of the observed IMI values.

# hypothesis tests at each observation
hyp.gradient <- predictions(mod.gradient,
 newdata = nd.gradient,
 vcov = vcov(mod.gradient, unconditional = T),
 type = "response",
 hypothesis = function(x){
 hypothesis.function(df.cf = x,
 null = "mu",
 stressor1 = "IMI",
 stressor2 = "TBZ",
 strategy = "rowwise")}) |>
 inferences(method = "simulation")

# determine categories
hyp.gradient <- hyp.gradient |>
 mutate(IMI.cat = case_when(IMI < quantile(IMI, probs = 0.25) ~ "1st quartile",
 IMI < quantile(IMI, probs = 0.5) ~ "2nd quartile",
 IMI < quantile(IMI, probs = 0.75) ~ "3rd quartile",
 IMI <= quantile(IMI, probs = 1) ~ "4th quartile"))

ggplot(data = hyp.gradient, aes(x = TBZ, y = estimate, color = IMI + 0.1)) +
 geom_point() +
 geom_hline(yintercept = 0, linetype = "dashed") +
 geom_errorbar(aes(ymin = conf.low, ymax = conf.high)) +
 #scale_x_continuous(transform = "log10") +
 facet_wrap(facets = vars(IMI.cat), scales = "free_y", nrow = 4) +
 labs(x = "TBZ", y = "Interaction estimate") +
 scale_color_viridis(trans = "log")


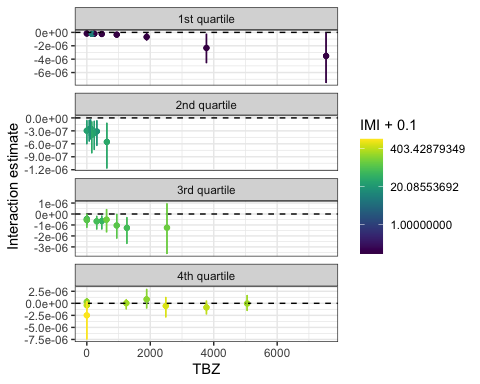


For the combination of low to medium concentrations of TBZ and IMI, we find many interaction estimates that indicate synergism. However, we should not rely on the pointwise confidence intervals. Again, we continue by calculating the average interaction effect and its uncertainty.

hyp.gradient.avg <- predictions(mod.gradient,
 newdata = nd.gradient,
 vcov = vcov(mod.gradient, unconditional = T),
 type = "response",
 hypothesis = function(x){
 hypothesis.function(df.cf = x,
 null = "mu",
 stressor1 = "IMI",
 stressor2 = "TBZ",
 strategy = "average")}) |>
 inferences(method = "simulation")

ggplot(hyp.gradient.avg, aes(x = term, y = estimate)) + geom_point(size = 3) +
 geom_errorbar(aes(ymin = conf.low, ymax = conf.high), width = 0.2) +
 facet_wrap(facets = vars(term), scales = "free") +
 geom_hline(aes(yintercept = 0), linetype = "dashed") +
 labs(x = "null model", y = "Interaction estimate (TBZ : IMI)")


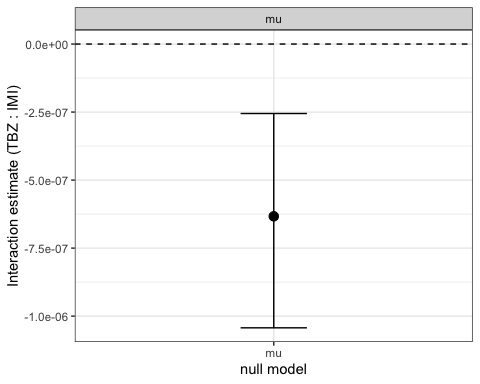


The average interaction estimate confirms the presence of a statistically significant interaction between the two toxicants. The joint effect on *Hyalella azteca* is more negative than expected from the multiplicative null model (synergism).

We end this analysis by evaluating if the interaction estimates are sensitive to step size for the approximation of the slope. This is easier to evaluate if we predict not at the observed values, but along a fine gradient for one toxicant (TBZ), and at specific values for the other (IMI).

ref.gradient2 <- datagrid(newdata = df.gradient,
 IMI = fivenum,
 TBZ = seq(0, 7541.2, length.out = 50))

for(i in c(1:10)){
 .DIFF1 <- range(df.gradient$IMI) |> diff() * 10^(-i)
 .DIFF2 <- range(df.gradient$TBZ) |> diff() * 10^(-i)

 .nd.gradient <- nd.generator(ref.gradient2,
 stressor1 = "IMI",
 stressor2 = "TBZ",
 diff = c(.DIFF1, .DIFF2))

 .hyp.gradient <- predictions(mod.gradient,
 newdata = .nd.gradient,
 vcov = vcov(mod.gradient, unconditional = T),
 type = "response",
 hypothesis = function(x){
 hypothesis.function(df.cf = x,
 null = "mu",
 stressor1 = "IMI",
 stressor2 = "TBZ",
 strategy = "rowwise")}) |>
 inferences(method = "simulation") |>
 mutate(DIFF1 = .DIFF1, DIFF2 = .DIFF2)

 if(i == 1) diff.choice <- .hyp.gradient else {
 diff.choice <- add_case(diff.choice, .hyp.gradient)}
 }

ggplot(data = diff.choice, aes(x = TBZ, y = estimate, color = IMI)) +
 geom_line(aes(group = IMI)) +
 geom_hline(yintercept = 0, linetype = "dashed") +
 geom_ribbon(aes(ymin = conf.low, ymax = conf.high, group = IMI), alpha = 0.2) +
 facet_wrap(facets=vars(DIFF1, DIFF2), scales = "free") +
 labs(x = "TBZ", y = "Interaction estimate") +
 scale_color_viridis()


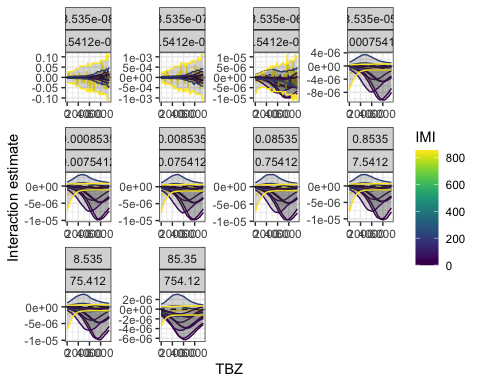
 The lowest step size in our data set that still produces stable confidence intervals corresponds to 0.00001 % of the stressor gradients.

# References

Beermann, A. J., Elbrecht, V., Karnatz, S., Ma, L., Matthaei, C., Piggott, J., & Leese, F. (2018). Multiple-stressor effects on stream macroinvertebrate communities: A mesocosm experiment manipulating salinity, fine sediment and flow velocity. *Science of The Total Environment, 610–611*, 961–971. <https://doi.org/10.1016/j.scitotenv.2017.08.084>

Greco, D. A., Arnott, S. E., Fournier, I. B., & Schamp, B. S. (2023). Effects of chloride and nutrients on freshwater plankton communities. Limnology and Oceanography Letters, 8(1), 48–55. <https://doi.org/10.1002/lol2.10202>

Raby, M., Maloney, E., Poirier, D. G., & Sibley, P. K. (2019). Acute Effects of Binary Mixtures of Imidacloprid and Tebuconazole on 4 Freshwater Invertebrates. *Environmental Toxicology and Chemistry, 38(5)*, 1093–1103. <https://doi.org/10.1002/etc.4386>

Wood, S. N. (2011). Fast stable restricted maximum likelihood and marginal likelihood estimation of semiparametric generalized linear models. *Journal of the Royal Statistical Society: Series B (Statistical Methodology), 73(1)*, 3–36. <https://doi.org/10.1111/j.1467-9868.2010.00749.x>

# Session information

sessionInfo()

## R version 4.4.1 (2024-06-14)
## Platform: aarch64-apple-darwin20
## Running under: macOS 15.5
##
## Matrix products: default
## BLAS: /Library/Frameworks/R.framework/Versions/4.4-arm64/Resources/lib/libRblas.0.dylib
## LAPACK: /Library/Frameworks/R.framework/Versions/4.4-arm64/Resources/lib/libRlapack.dylib; LAPACK version 3.12.0
##
## locale:
## [1] en_US.UTF-8/en_US.UTF-8/en_US.UTF-8/C/en_US.UTF-8/en_US.UTF-8
##
## time zone: Europe/Berlin
## tzcode source: internal
##
## attached base packages:
## [1] grid stats graphics grDevices utils datasets methods
## [8] base
##
## other attached packages:
## [1] ggpubr_0.6.0 gridExtra_2.3 gtable_0.3.6
## [4] latex2exp_0.9.6 viridis_0.6.5 viridisLite_0.4.2
## [7] marginaleffects_0.27.0 DHARMa_0.4.6 mgcv_1.9-3
## [10] nlme_3.1-165 lubridate_1.9.3 forcats_1.0.0
## [13] stringr_1.5.1 dplyr_1.1.4 purrr_1.0.4
## [16] readr_2.1.5 tidyr_1.3.1 tibble_3.3.0
## [19] ggplot2_3.5.2 tidyverse_2.0.0
##
## loaded via a namespace (and not attached):
## [1] tidyselect_1.2.1 farver_2.1.2 fastmap_1.2.0 GGally_2.2.1
## [5] promises_1.3.0 digest_0.6.36 timechange_0.3.0 mime_0.12
## [9] lifecycle_1.0.4 magrittr_2.0.3 compiler_4.4.1 rlang_1.1.6
## [13] tools_4.4.1 utf8_1.2.6 yaml_2.3.9 data.table_1.17.4
## [17] ggsignif_0.6.4 knitr_1.48 labeling_0.4.3 bit_4.0.5
## [21] plyr_1.8.9 RColorBrewer_1.1-3 gap.datasets_0.0.6 abind_1.4-5
## [25] gamm4_0.2-6 KernSmooth_2.23-24 miniUI_0.1.1.1 withr_3.0.2
## [29] xtable_1.8-4 scales_1.4.0 iterators_1.0.14 MASS_7.3-61
## [33] insight_1.3.0 mvtnorm_1.2-5 cli_3.6.5 rmarkdown_2.27
## [37] crayon_1.5.3 generics_0.1.4 rstudioapi_0.16.0 tzdb_0.4.0
## [41] minqa_1.2.7 splines_4.4.1 parallel_4.4.1 matrixStats_1.4.1
## [45] vctrs_0.6.5 boot_1.3-30 Matrix_1.7-0 carData_3.0-5
## [49] car_3.1-2 hms_1.1.3 rstatix_0.7.2 bit64_4.0.5
## [53] qgam_1.3.4 foreach_1.5.2 gap_1.6 glue_1.8.0
## [57] nloptr_2.1.1 ggstats_0.7.0 codetools_0.2-20 stringi_1.8.7
## [61] later_1.3.2 lme4_1.1-35.5 pillar_1.10.2 htmltools_0.5.8.1
## [65] R6_2.6.1 Rdpack_2.6.1 doParallel_1.0.17 vroom_1.6.5
## [69] evaluate_0.24.0 shiny_1.9.1 lattice_0.22-6 highr_0.11
## [73] rbibutils_2.3 backports_1.5.0 broom_1.0.6 httpuv_1.6.15
## [77] Rcpp_1.0.14 checkmate_2.3.1 mgcViz_0.1.11 xfun_0.52
## [81] pkgconfig_2.0.3
